# Endoscopic surgery versus various open approaches in esthesioneuroblastoma: a systematic review of the literature

**DOI:** 10.3389/fonc.2025.1512771

**Published:** 2025-05-28

**Authors:** Michael Koch, Matthias Balk, Sven Schlaffer, Moritz Allner, Heinrich Iro, Sarina K. Müller

**Affiliations:** ^1^ Department of Otorhinolaryngology and Head and Neck Surgery, University of Erlangen–Nuremberg, Erlangen, Germany; ^2^ Department of Neurosurgery, University of Erlangen–Nuremberg, Erlangen, Germany

**Keywords:** endoscopic, transcranial, craniofacial, transfacial, open, surgery, esthesioneuroblastoma, olfactorius neuroblastoma

## Abstract

**Objective:**

Esthesioneuroblastoma (ENB) is treated using several open surgery (OpS) methods, with or without endoscopic assistance ( ± E-ass) or endoscopic surgery (ES). This systematic review compared the results with various approaches using OpS ± E-ass and ES.

**Data sources:**

A systematic PubMed/Medline search was conducted for the period 1990–2023.

**Review methods:**

Keywords were “esthesioneuroblastoma” or “olfactory neuroblastoma” and “surgery,” “surgical,” “resection,” “approach,” “open,” and “endoscopic.” Studies/case series and case reports were included. Results with OpS ± E-ass (stratified into various approaches) were compared with ES results. Parameters assessed were follow-up period, frequencies of advanced tumor stages, Hyams grade III–IV tumors, negative margins/gross total resection, postoperative complication rates, preoperative/postoperative radiation therapy/chemotherapy, primary tumor progression, and frequency of/time to first recurrence.

**Results:**

A total of 88 studies/case series or single cases/case reports (SC/CR) with results after OpS ± E-ass (850 cases) and 84 with results after ES (584 cases) were included. Compared with OpS ± E-ass, after ES, the average follow-up was significantly shorter (*p*=0.048) and mean crude disease-free survival (DFS) significantly better (studies/case series, *p*=0.0001; SC/CR, *p*=0.001). Compared with OPS ± E-ass, after ES, significantly fewer advanced tumors were treated (studies/case series, *p*=0.0001; SC/CR, *p*=0.001); negative margins were significantly less frequent (studies/case series, *p*=0.009); surgical complications were less frequent (studies/case series, *p*=0.022); less radiation therapy (studies/case series, *p*=0.043) and/or chemotherapy (SC/CR, *p*=0.022) was performed; and recurrences were noted significantly less often (studies/case series, *p*=0.0001; SC/CR, *p*=0.034). Among OpS ± E-ass, craniofacial resection ± E-ass showed most significant differences from ES.

**Conclusions:**

These data support that ES can be regarded as the surgical method of first choice in less advanced ENB but may also be a good choice in carefully selected advanced ENB.

## 1 Introduction

The treatment for esthesioneuroblastoma (ENB) consists of complete surgical resection and adjuvant therapy (1–9). The literature shows that there has been a shift from open surgery approaches (OpS) to endoscopic surgery (ES). Open bicoronar/transcranial resection (BCR/TCR), craniofacial resection (CFR), and transfacial resection (TFR) were regarded as the gold standard in publications up to the 2000s (10–12). BCR/TCR, CFR, or TFR with endoscopic assistance (BCR/TCR+E-ass, CFR+E-ass, and TFR+E-ass) was introduced in the late 1990s and early 2000s to reduce invasiveness and morbidity (3, 13, 14).

ES has been performed since the beginning of this century, and the results have been published in numerous reports (3, 15, 16). Tumor stage is regarded as a significant prognostic factor, but there is no universally accepted staging system (17, 18). The tumor classification systems proposed by Kadish (19)/Morita (20) and Dulguerov and Calcaterra (21) have most often been assessed. Histopathological classification based on the Hyams grading is now increasingly being recognized as an important prognostic factor (22–24).

The aim of this study was to carry out a literature review to compare the results and outcome in patients undergoing OpS ± E-ass, stratified according to BCR/TCR ± E-ass, CFR ± E-ass, and TFR ± E-ass, and patients receiving ES, relative to known prognostic factors.

## 2 Methods

**Figure 1:**
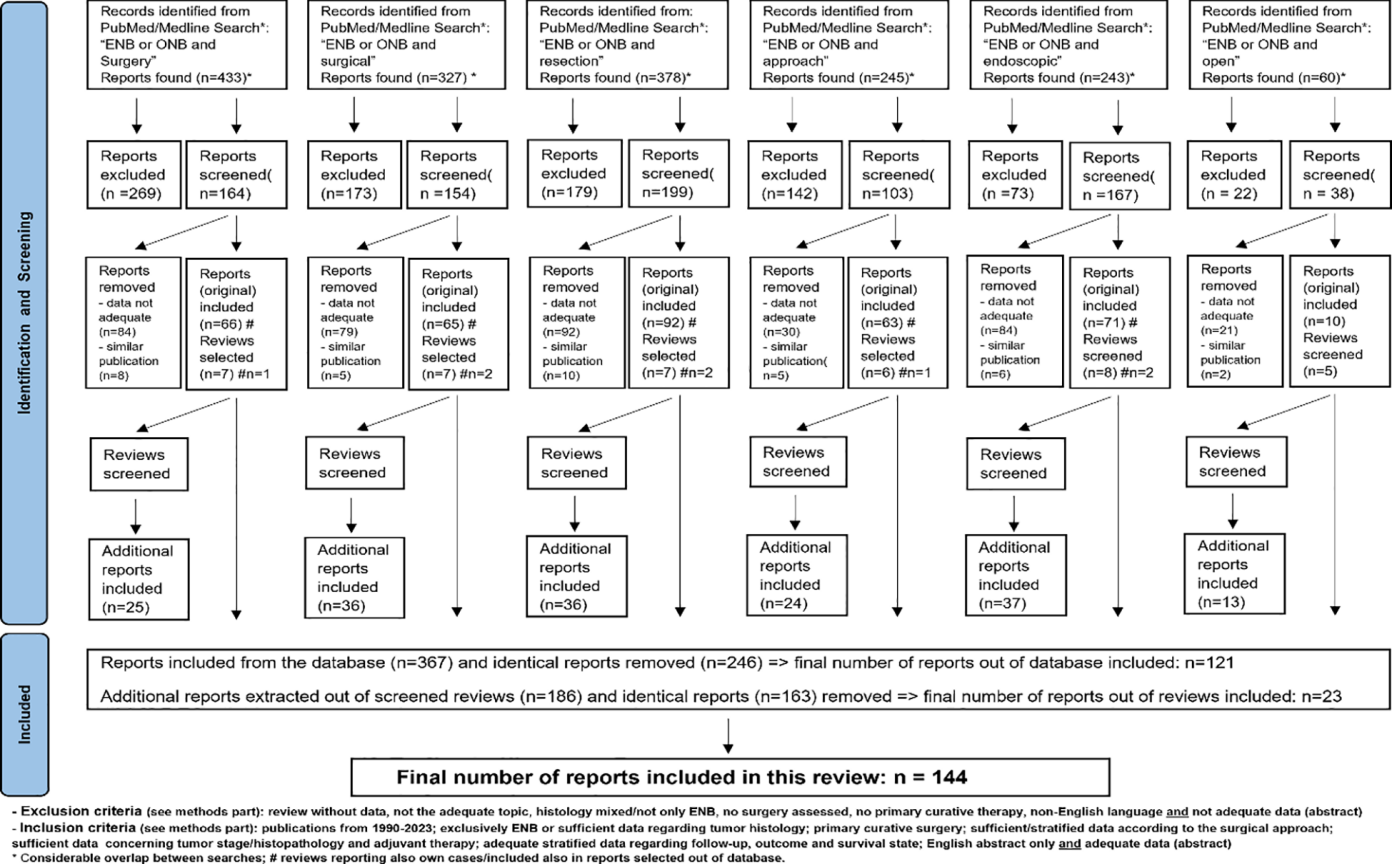
Selection process of studies, case series and single cases/case reports for this systematic review.

A literature review for the period 1990–2023 was performed, using the PubMed/Medline database to search for publications reporting results after surgery for ENB with OpS ± E-ass and ES. The keywords (in the title or abstract each) used were: “esthesioneuroblastoma” OR “olfactory neuroblastoma” AND “surgery” or “surgical” or “resection” or “approach” or “endoscopic” or “open.” The systematic review was conducted considering the PRISMA criteria Flow diagram (**Figure 1**).

Available reviews or meta-analyses were also analyzed for publications cited discussing surgery in pediatric ENB (7), ES (16), OpS (12), and comparisons of OpS ± E-ass and ES (3, 25–27). Besides studies and case series (STUD/CS), also case reports (CR, summarized together with single cases described in STUD/CS as “SC/CR”) were selected, since many of these describe treatment for advanced ENB and/or ENB associated with specific symptoms and/or unusual locations (28, 29).

Only publications dealing exclusively with ENB and/or that provided sufficient data or results of interest regarding surgical treatment were included. Reports that did not focus on ENB alone but included sufficient and stratified data of interest were also considered. To be suitable for inclusion, STUD/CS had to fulfill criteria (see also the PRISM flow diagram).

Inclusion criteria were in detail: publications published between 1990 and 2023; management of ENB exclusively and/or at least sufficient data regarding tumor histology in publications dealing with various tumors; surgical treatment for ENB with curative intent; publications providing clear definition/stratification of the data relative to the surgical approach, tumor stage/histopathology, and adjuvant therapy; publications that report adequate stratified data regarding follow-up; publications providing adequate follow-up/outcome data/survival data; and publications written in English language or providing an abstract written in English and simultaneously providing adequate data in the abstract. Only the most recent publication was selected if several follow-up reports were published by one group.

Exclusion criteria were in detail: reports that did not provide stratified data regarding the surgical approach or no sufficient data regarding follow-up times or the survival status; publications not dealing with the surgical therapy of ENB; publications dealing with mixed tumors in which no histologically proven ENB could be clearly assigned to the parameters investigated; and publications not written in English language or at least providing an abstract written in English, which includes simultaneously adequate data of interest.

A meticulous review was carried out in all STUD/CS to sample as many as much stratified data of interest as possible, also by calculating these from the materials provided within the reports (e.g., tables).

The parameters of interest assessed in the present study were type of OpS ± E-ass (BCR/TCR ± E-ass, CFR ± E-ass, and TFR ± E-ass) and ES; number of patients operated on, number of conversions from ES to OpS ± E-ass; follow-up period; crude/actuarial survival data; number of patients with advanced tumor-stages (Kadish/Morita and/or Dulguerov and Calcaterra); number of Hyams grade III–IV tumors; frequency of negative margins (NM), gross total (GTR), or complete resection (CoR: NMs plus GTR); frequency of postoperative complications; frequency/dosage of preoperative and/or postoperative radiation therapy (RT) and/or chemotherapy (ChT); frequency of tumor progression; and first recurrence including time after surgery. Crude/actuarial survival was given as provided in the publications, as overall (OS), disease-specific (DSS), disease-free (DFS), recurrence-free (RFS), progression-free (PFS), and local recurrence–free survival (LRFS).

To indicate how many STUD/CS or SC/CR provided data concerning specific parameters, the term “reports provided data” is used, with the abbreviation “RPD.”

SPSS Statistics for Windows, version 26, was used for analysis (IBM Corporation, Armonk, New York, USA). Although the data in the tables were stratified for TCR/BCR, CFR, or TFR with and without E-ass, all statistical calculations were performed for summarized data regarding TCR/BCR, CFR, or TFR regardless if it was performed with or without E-ass. The average, median, and range of the (mean) values were calculated. Differences/associations between the groups were calculated for continuous and categorical variables using the Mann–Whitney U exact test or chi-square exact test, respectively. A comparison of groups was made if at least five values per group were reported. The significance level was set at *p ≤* 0.05.

## 3 Results

A total of 144 STUD/CS or CRs, including 1,434 patients, were identified and included in this review (Flow Diagram). Due to the huge number of publications and data, details are summarized in **Supplementary Tables S1**-**S5B**.

### 3.1 Open surgery

A total of 850 patients were extracted out of 88 STUD/CS and CRs selected, published from 1992 to 2023. The results of various OpS ± E-ass and/or ES procedures were reported in 25 STUD/CS.

#### 3.1.1 BCR/TCR ± E-ass

A total of 18 STUD/CS or CRs including 96 patients published from 1992 to 2023 were found. BCR/TCR-E-ass was described in eight STUD/CS (2, 30–36), BCR/TCR+E-ass in nine (28, 37–44), and BCR/TCR ± E-ass was reported in one (45) (**Supplementary Tables S1A, B**). Results with other OpS ± E-ass and/or ES procedures were reported in six STUD/CS (2, 28, 32, 36, 38, 45).

#### 3.1.2 Case series/studies

Seven STUD/CS including 85 patients were published, the mean follow-up times was 53.8 (range 22–84) months. Crude OS was 87.5–100%, DSS 66.7–100%, and DFS 73.3–100% (maximum 5 RPD). Advanced tumors were present in 13.3–100% and in ≥50% in four STUD/CS. High-grade tumors were present in 14.3–86.7% (5 RPD). NMs were achieved in 71% (two RPD) and 100%, CoR in 82%-100% (4 RPD). Postoperative complications were observed in 0–42.3% of all cases. RT was administered in 40–100% of patients, with a dose range of 50–65Gy (3 RPD). ChT was performed in 0–33.3% (6 RPD). Primary tumor-progression was reported in one study (6.7%) (2). First recurrence occurred after 1–78 months in 12.5–66.7% of patients (7 RPD), in one report after a mean of 82.1 months (34), and in another >5 years in 29% of the patients (45) (**Table 1a**; **Supplementary Table 1**).

**Table 1A:** Summary of results: average of the mean of the parameters investigated in studies/case series and stratified relative to OpS ± E-ass (BCR/TCR ± E-ass; CFR ± E-ass; TFR ± E-ass).

Parameter	Surgical group				
	BCR/TCR ± E-ass (n=7)	CFR ± E-ass (n=33)	TFR ± E-ass (n=10)	OpS ± E-ass total (n=50)	ES (n=44)
Average of follow-up periods (months, mean, median, range) ^+^	53.81 (M 62.3; R 22–84)	61.38 (M 58.5, R 13–107.3)	76.57 (M 75.8; R 33.5–118.5)	63.28 (M 63.7; R 13–118.5)	51.81 (M 44.85, R 12.5–125.2)
Total range of reported follow-up times (months)^+^	1.3–252	0.1–360	2–225	0.1–360	3–242
Crude survival-OS ^+^ (%, mean, median, range)	94.6 (M 95.4, R 87.5–100)	60.3 (M 57.1; R 57.1–66.7)	62.8 (M 62.8; R 33.3–92.3)	76.1 (M 87.5, R 33.3–100)	98.1 (M 100, R 60–100)
Crude survival-DSS ^+^ (%, mean, median, range)	86.2 (M 90.9; R 66.7–100)	73.1 (M 71.4, R 50–100)	82.3 (M 84.6, R 60–100)	77.4 (M 72.4, R 50–100)	100 (M 100, R 100–100)
Crude survival-DFS ^+^ (%, mean, median, range)	81.5 (M 75, R 73.3–100)	66.9 (M 66, R 30–100)	73.7 (M 76.9, R 33.3–100)	70.4 (M 73.3, R 30–100)	96.5 (M 100, R 76.9–100)
Advanced tumors (%, mean, median, range) ^+^	58.9 (M 53.3, R 13.3–100)	72.0 (M 72.7, R 0–100)	31.4 (M 22.5, R 0–66.7)	61.6 (M 66.7, R 0–100)	37.2 (M 33.3, R 0–100)
Hyams high-grade tumors (% cases/study or case series, mean, range) ^+^	54.9 (M 66.7, R 14.3–86.7)	39.1 (M 36.6, R 20–66.7)	17.7 (M 20, R 0–33.3)	40.1 (M 36.6, R 0–86.7)	27.8 (M 25; R 0–92.6)
Hyams high-grade tumors ≥50% of cases in studies/case series ^+^	60.0	25.0	0	33.3	25.0
Resection status - negative margins (NM) (%, mean, range) ^+^	85.5 (M 85.5; R 71–100) *	71.2 (M 75, R 14.3–100)	71.1 (M 80, R 33.3–100)	72.6 (M 75, R 14.3–100)	88.5 (M 92.3, R 50–100) *
Resection status − gross total resection (GTR; %, mean, range) ^+^	70.3 (M 100, R 11–100) *	69.9 (M 25; R 25–100)	75 (M 75; 50–100)	71.3 (M 92.3; R 11−100)	74.4 (M 100; 7.7−100)*
Complete resection total (NM or GTR/patients total; %, mean, range) ^+^	95.5 (M 100, R 82–100) *	75.7 (M 78.6, R 14.3–100)	84.3 (M 96.1, R 33.3–100)	80.9 (M 84.6, R 14.3–100)	89.0 (M 100, R 50–100) *
Postoperative complications (%, mean, range) ^+^	25.0 (M 30, R 0–42.3)	19.5 (M 16.7, R 0–62.5)	12.6 (M 7.7, R 0–40)	19.4 (M 18.4, R 0–62.5)	10.5 (M 5.7, R 0–60)
Pre-/postoperative RT (%, mean, range) ^+^	81.6 (M 80.0, R 40–100)	76.8 (M 81.3, R 0–100)	69.1 (M 75, R 0–100)	76.0 (M 80.0, R 0–100)	83.8 (M 90.3, R 0–100)
Pre-/postoperative ChT (%, mean, range) ^+^	10.6 (M 6.7, R 0–33.3)	32.0 (M 23.1, R 0–100)	10.7 (M 3.9, R 0–33.3)	24.7 (M 16.7, R 0–100)	18.9 (M 0, R 0–88.9)
First recurrences (%, mean, range) ^+^	33.8 (M 28.6, R 0–66.7)	35.1 (M 33.3, R 0–83.3)	23.4 (M 15.4, R 0–61.5)	32.6 (M 33.3, R 0–83.3)	14.8 (M 10, R 0–100%)
Time to first recurrences (range, months) ^+^	1–78 ^#^	1–312	2–84	1–312	3–168
Primary tumor progression (%)	6.7%	14.9 (12.5–16.7)	0/7.7 ~	12.8 (6.7–16.7)	0

^+^If available: not in all studies reported; *71% NM and 11% GTR summarize to an 82% complete resection rate in one and 7.7% and 92.3% in another publication; ^#^a mean of 82.1 months reported in the study of Ward et al. (34); ~one case after TFR (7.7%) out of a case series (21).

BCR, bicoronal resection; CFR, craniofacial resection; ChT, chemotherapy; DFS, disease-free survival; DSS, disease-specific survival; E-ass, endoscopy-assisted; ES, endoscopic surgery; GTR, gross total resection; NM, negative margins; OpS, open surgery OS, overall survival; RT, radiotherapy; TCR, transcranial resection; TFR, transfacial resection.

#### 3.1.3 Single cases/case reports

Two single cases were described as part of STUD/CS (32, 38), and nine cases were included in CRs (28, 30, 33, 35, 39, 40, 42–44). The mean follow-up time was 35.7 (range, 6–102) months. Crude OS and DSS rates were 100% each, DFS of 81.8%. Kadish stage C tumors were treated in 90.0% of the patients. A high-grade tumor was described in 16.7% (6 RPD). NMs were achieved in 50% (4 RPD) and CoR in 77.8% (9 RPD). Postoperative complications were observed in 40%, and RT was administered in 90.9% of all cases, with a dosage range of 53.2–60 Gy (5 RPD); 45.5% received ChT. Palliative ChT and biological agents were administered in one CR for regional and distant metastases, which were detected after 2 months, apparently difficult to distinguish from tumor-progression (44) (**Table 1b**; **Supplementary Table S1**).

**Table 1b:** Summary of results: average of the mean of the parameters investigated in single cases in case series/case reports, and stratified relative to OpS ± E-ass (BCR/TCR ± E-ass; CFR ± E-ass; TFR ± E-ass; total) and endoscopic surgery (ES).

Parameter	Surgical group				
	BCR/TCR ± E-ass (n=11)	CFR ± E-ass (n=16)	TFR ± E-ass (n=11)	OpS ± E-ass total (n=38)	ES (n=40)
Follow-up period (months, mean, median, range) ^+^	35.73 (M 14.0, R 6–102)	33.31 (M 16, R 3–134)	21.6 (M 21.5, R 10–36)	30.86 (M 18, R 3–134)	32.11 (M 24, R 3–120)
Crude survival (%)^+^-OS	100	87.5	81.8	89.5	97.5
Crude survival (%)^+^-DSS	100	87.5	81.8	89.5	97.5
Crude survival (%)^+^-DFS	81.8	87.5	63.6	78.9	97.5
Advanced tumors (%) ^+^	90.9	87.5	30.0	73.0	35
Hyams high-grade tumors (%)^+^	16.7	60.0	42.9	43.5	35.0
Resection status^+^-NM (%)	50	33.3	100	40.9	66.7
Resection status^+^-GTR (%)	55.5	80	100	91.7	100
Resection status (%)^+^ - NM or GTR (%)	77.8	53.3	100	69.0	78.1
Postoperative complications (%) ^+^	40.0	21.4	25	28.1	5.6
Pre-/postoperative RT (%) ^+^	90.9	85.7	72.7	83.3	67.5
Pre-/postoperative ChT ( %) ^+^	45.5	42.9	9.1	33.3	10.0
First recurrences (%) ^+^	27.3	18.8	36.4	26.3	7.5
Time to first recurrences (months) ^+^	2–29	1-60	12-13	1-60	5–24
Primary tumor progression (%)	0/9.1 ~	6.25	0	2.6/5.3 ~	0

^+^If available: not in all studies reported; ~one case report after TCR/BCR (44).

BCR, bicoronal resection; CFR, craniofacial resection; ChT, chemotherapy; DFS, disease-free survival; DSS, disease-specific survival; E-ass, endoscopy-assisted; ES, endoscopic surgery; GTR, gross total resection; NM, negative margins; OpS, open surgery OS, overall survival; RT, radiation therapy; TCR, transcranial resection; TFR, transfacial resection.

### 3.2 CFR ± E-ass

A total of 49 STUD/CS and CRs including 628 patients, published from 1992 to 2021, were found. CFR-E-ass was evaluated in 35 STUD/CS (18, 21, 22, 46–76), CFR+E-ass in 12 (18, 74, 76–84), and CFR ± E-ass in two studies (14, 85). Results for CFR and several OpS ± E-ass and/or ES procedures were reported in 20 STUD/CS (18, 21, 38, 47, 48, 59–61, 63, 65, 66, 68, 71, 72, 74–77, 85).

#### 3.2.1 Case series/studies

A total of 33 STUD/CS including 612 patients were published. The average of the mean/median of follow-up periods was 61.4 (range, 13–107.3) months (28 RPD). Crude survival for OS was 57.1%–66.7% (3 RPD), DSS was 50%–100% (15 RPD), and DFS was 30%–100% (25 RPD). The actuarial 5-year survival rates (11 RPD) were 60%–95.2% for OS (54, 62, 64, 65, 72, 79, 84), 77%–82.6% for DSS (54, 62), 28.6%–86.5% for DFS (18, 58, 64, 65, 71), and 49%–64.2% for RFS (62, 84). The 5-year local control rate was 100% (79), and 10-year survival rates were 42%–93% for OS (54, 65, 72, 84), 53% for DSS (54), and 57.1% for DFS (65). One study reported a 15-year DFS rate of 82.6% (58). Advanced tumors were treated in 0%–100% of patients and were present in >50% of cases in 90.9% (28/31) of STUD/CS. With Hyams grading (8 RPD), high-grade tumors were noted in 20%–66.7%. NMs and CoRs were achieved in 14.3%–100% each (13 and 15 RPD). Postoperative complications occurred with a frequency of 0%–62.5% (19 RPD). RT was administered in 0%–100% of cases (31 RPD), with a dosage range of 18–90 Gy (19 RPD). ChT was administered in 0%–100% of all cases (31 RPD). Primary tumor progression was observed in three STUD/CS (12.5%–16.7% of cases). First recurrences after surgery were observed in 9.1%–83.3% of the cases (30 RPD) after time intervals ranging from 1 to 312 months (**Table 1a**; **Supplementary Table S2**).

#### 3.2.2 Single cases/case reports

Two single cases were described in STUD/CS (74, 76), while CRs described 14 cases (52, 56, 57, 67, 69, 70, 73, 78, 80–83, 86, 87). The mean follow-up period was 33.3 (range, 3–134) months, and the mean OS/DSS/DFS rate was 87.5% each. Kadish stage C lesions were found in 87.5% of all cases and high-grade tumors in 60% (10 RPD). NMs were reported in 33.3 (12 RPD) and CoR in 53.3% (15 RpD). Postoperative complications were noted in 21.4% of all cases. RT was performed in 85.7% and ChT in 42.9% of patients (14 RPD each). First recurrences were observed in three cases. Tumor progression occurred in one case (67). Another patient had a recurrence and signs of an unfavorable tumor (Kadish stage C, high grade, and positive margins) (74). Both patients died (**Table 1b**; **Supplementary Table S2**).

### 3.3 TFR ± E-ass

A total of 21 STUD/CS including 82 patients were published from 1992 to 2018. Results after TFR-E-ass were published in 12 STUD/CS (21, 47, 48, 59, 60, 68, 72, 88–92) and results after TFR+E-ass in nine (66, 74, 93–99).

#### 3.3.1 Case series/studies

A total of 10 STUD/CS were published including 71 patients with results after TFR ± E-ass (21, 47, 48, 60, 66, 68, 72, 88, 94, 95). The average of the mean follow-up times was 76.6 (range, 33.5–118.5) months (8 RPD). Crude survival (maximum of 9 RPD) was 33.3%–92.3% for OS, 60%–100% for DSS, and 33.3%–100% for DFS. Advanced-stage tumors were present in >50% of the patients in three STUD/CS (10 RPD). Hyams high-grade tumors were present in 0%–33.3% (3 RPD). NMs and CoR were achieved in 33.3%–100% each (5 and 6 RPD). Postoperative complications were reported in 0%–40% of cases (6 RPD). RT was administered in 80% of STUD/CS in 53.8%–100% of cases (9 RPD). The dosage and/or range administered (50–65 Gy) were reported in four STUD/CS. ChT was administered in 0%–33.3% of the patients (10 RPD). Primary tumor progression could be suggested in one case after recurrence occurred after a short period in connection with “dead of disease” (DOD) status (7.7%) (21). First recurrences were described in 0%–61.5% of cases after periods ranging from 2 to 84 months (9 RPD, **Table 1a**; **Supplementary Table S3**).

#### 3.3.2 Single cases/case reports

Results after TFR ± E-ass were reported in two STUD/CS in one case each (59, 74) and in nine CRs (89–93, 96–99). The average follow-up was 21.6 (range, 10–36) months (10 RPD). The crude survival rates (maximum of 11 RPD) were 81.8% for OS and DSS and 63.6% for DFS. Advanced tumors were present in 30% (10 RPD) and high-grade tumors in 42.9% (7 RPD). NMs and CoR were achieved in all cases reported (3 and 5 RPD). A postoperative complication was noted in 25% (8 RPD). RT was administered in eight cases (11 RPD), and the dosage was described in four cases (all 60 Gy). One patient declined RT and died (93). One patient received ChT. No tumor progression was noted. First recurrences were observed in 36.4% of all cases after a period of 12–13 months (11 RPD, **Table 1b**; **Supplementary Table S3**).

### 3.4 Various OpS ± E-ass

A range of combined and/or staged surgery (same case) or mixed OpS ± E-ass were described in two STUD/CS and three CRs including 44 patients but were not intensely evaluated in this review, as the data were not stratified to the surgical approach (**Supplementary Table S4**) (100–104).

### 3.5 Endoscopic surgery

A total of 84 STUD/CS or CRs including 584 patients, published from 2000 to 2023, were selected (2, 14, 18, 28, 29, 32, 36, 38, 45, 59, 61, 63, 65, 66, 68, 71, 74 ,75, 77, 85, 105–167). Results after OpS ± E-ass and ES were described in 20 publications (2, 14, 18, 28, 29, 32, 36, 38, 45, 59, 61, 63, 65, 66, 68, 71, 74, 75, 77, 160).

A total of 44 STUD/CS including 544 patients with results after ES were published (2, 14, 18, 28, 29, 32, 36, 38, 45, 61, 65, 66, 68, 71, 74–76, 85, 106, 111, 113, 115, 116, 118, 119, 123, 124, 127, 129, 133, 135–137, 140, 141, 143–145, 149, 152, 153, 155, 157, 160).

The average of the mean follow-up times was 51.8 (range, 12.5–125.2) months (40 RPD). Crude survival rates (maximum of 27 RPD) were 60%–100% for OS, 76.9%–100% for DFS, and 100% for DSS. The actuarial 5-year survival was 84.6%–100% for OS (65, 119, 133, 137, 152, 153, 155, 160), 100% for DSS (129, 155), 50%–100% for DFS (18, 65, 71, 74, 75, 129, 133, 137, 152, 153, 160), 75%–92.9% for RFS (133, 155), and 38.5% for PFS (85). The 10-year survival was 87.5%–100% for OS (65, 85, 137) and 75.6% and 90% for DFS (65, 137).

#### 3.5.1 Case series/studies

Advanced stage tumors were present in 0%–100% (41 RPD), and in 47.7% of the STUD/CS, ≥50% of the patients treated had advanced tumor stages. High-grade tumors were present in 0%–92.6% of cases (21 RPD). NMs (27 RPD) and CoR (31 RPD) were achieved in 50%–100% of cases each. Interestingly, conversion from ES to OpS ± E-ass was described only in publications in individual cases up to the year 2010 (32, 111, 113, 123) but was no longer reported later. Postoperative complications occurred in 0%–60% (28 RPD). RT was performed in 41 STUD/CS in 33.3%–100% of the patients (42 RPD). The dosage range was 24–66Gy (21 RPD). ChT was administered in 22 studies in 7.7%–88.9% (42 RPD). Primary tumor progression was not observed. First recurrences (40 RPD) were reported to occur with a mean rate of 14.8% per STUD/CS (range, 0%–100%) and after time intervals of 3–168 months (16 RPD) (**Table 1a**; **Supplementary Table 5A**).

#### 3.5.2 Single cases/case reports

Results after treatment of only one case (SC/CR) with ES were reported in 40 publications. In three of these, ES was part of STUD/CS that also included OpS ± E-ass (59, 63, 77), and 37 were CRs in which specific situations (e.g., sense of smell preservation and specific histopathology), treatment of advanced tumors, tumors with an atypical/ectopic location, or tumors presenting with unusual symptoms were addressed (105, 107–110, 112, 114, 117, 120–122, 125, 126, 128, 130–132, 134, 138, 139, 142, 146–148, 150, 151, 154, 156, 158, 159, 161–167).

The mean follow-up period was 32.1 (range, 3–120) months. The crude data for OS/DSS/DFS showed 97.5% each. Kadish stages C/D were noted in 35% of the lesions, and Hyams grade III/IV tumors were present in 35.7% (20 RPD). NMs were achieved in 66.7% and CoR in 78.1% of cases (24 and 32 RPD). Postoperative complications were reported in 5.6% (36 RPD). RT was administered in 67.5% of all patients and ChT in 10%. No tumor progression was noted, but first recurrences were observed in 7.5% after 5–24 months. The only patient who died had a Kadish-C, high-grade ENB with a distant recurrence after 5 months (130) (**Table 1b**; **Supplementary Table S5B**).

### 3.6 Comparison of OpS ± E-ass and ES

The results of this review, classified relative to STUD/CS and SC/CR and comparing OpS ± E-ass (BCR/TCR ± E-ass, CFR ± E-ass, and TFR ± E-ass) and ES, are summarized in **Tables 1a**, **b** and **2a**, **b**.

**Table 2a:** Statistics for the parameters investigated in studies/case series: comparison of mean values for parameters in OpS ± E-ass (BCR/TCR ± E-ass, CFR ± E-ass, and TFR ± E-ass) and endoscopic surgery (ES).

Compare of OpS stratified to various types of OpS with ES and of all types of OpS with ES	BCR/TCR ± E-ass vs. ES	CFR ± E-ass vs. ES	TFR ± E-ass vs. ES	OpS ± E-ass vs. ES
Studies/case series				
Average for mean follow-up period/study ^#^	n.s. (*P* = 0.715)	n.s. (*P* = 0.116)	***p* = 0.034**	***p* = 0.048**
OS rates/study ^# ~^	n.n. ^+^	n.n. ^+^	n.n. ^+^	***p* = 0.0001**
NED/DFS rates/study ^# ~^	***p* = 0.013**	***p* = 0.0001**	***p* = 0.047**	***p* = 0.0001**
DSS rates/study ^# ~^	***p* = 0.035**	***p* = 0.0001**	n.n. ^+^	***p* = 0.0001**
Frequency of advanced tumors/study ^#^	n.s. (*p* = 0.131)	***p* = 0.0001**	n.s. (*p* = 0.658)	***p* = 0.0001**
Studies with advanced tumors: ≥ 50% of all cases (yes vs. no) *	n.s. (*p* = 0.419)	***p* = 0.0001**	n.s. (*p* = 0.483)	***p* = 0.006**
Hyams grading III–IV frequency/study	*n.s. (p = 0.057)*	n.s. (*p* = 0.153)	n.n.^+^	*n.s. (p = 0.064)*
Hyams grading III–IV: ≥ 50% of all cases (yes vs. no) *	n.s. (*p* = 0.287)	n.s. (*p* = 1.0)	n.n. ^+^	n.s. (*p* = 0.397)
Negative margins rate/study ^#^	n.n. ^+^	***p* = 0.008**	n.s. (*p* = 0.201)	***p* = 0.009**
Complete resection rate/study (NM or GTR) ^#^	n.n. ^+^	***p* = 0.036**	n.s. (*p* = 0.715)	n.s. (*p* = 0.234)
Surgical complication rates/study ^#^	***p* = 0.022**	***p* = 0.046**	n.s. (*p* = 0.809)	***p* = 0.022**
Surgical complication (yes vs. no) *	n.s. (*p* = 0.203)	n.s. (*p* = 0.226)	n.s. (*p* = 1.0)	n.s. (*p* = 183)
RT % of patients/study ^#^	n.s. (*p* = 0.769)	*n.s. (p = 0.065)*	*n.s. (p = 0.091)*	***p* = 0.043**
RT (yes vs. no) *	n.s. (*p* = 1.0)	n.s. (*p* = 1.0)	n.s. (*p* = 0.313)	n.s. (*p* = 1.0)
ChT % of patients/study ^#^	n.s. (*p* = 0.964)	***p* = 0.041**	n.s. (*p* = 0.689)	n.s. (*p* = 0.175)
ChT (yes vs. no) *	n.s. (*p* = 0.671)	***p* = 0.029**	n.s. (*p* = 1.0)	*n.s. (p = 0.084)*
Recurrence % of patients/study ^#^	***p* = 0.036**	***p* = 0.0001**	n.s. (*p* = 0.468)	***p* = 0.0001**
Recurrence (yes vs. no) *	n.s. (*p* = 0.215)	***p* = 0.020**	n.s. (*p* = 1.0)	***p* = 0.037**

^#^Mann–Whitney *U*-test/Fisher’s exact test; *chi-square exact test; ^+^no statistics: too few cases per group; ~Values were calculated from (raw) material in reports with variable follow-up times.

BCR, bicoronal transection; CFR, craniofacial resection; ChT, chemotherapy; DFS, disease-free survival; DSS, disease-specific survival; ES, endoscopic surgery; NED, no evidence of disease; OpS, open surgery; OS, overall survival; RT, radiation therapy; TCR, transcranial resection; TFR, transfacial resection. Bold letters/values should highlight significant results.

**Table 2b:** Statistics for the parameters investigated in single cases in case series/case reports): comparison of mean values for parameters in OpS ± E-ass (BCR/TCR ± E-ass, CFR ± E-ass, and TFR ± E-ass) and endoscopic surgery (ES).

Compare of OpS stratified to various types of OpS with ES and of all types of OpS with ES	BCR/TCR ± E-ass vs. ES	CFR ± E-ass vs. ES	TFR ± E-ass vs. ES	OpS ± E-ass vs. ES
Single cases in case series/case reports				
Average of mean follow-up ^#^	n.s. (*p* = 0.580)	n.s. (*p* = 0.246)	n.s. (*p* = 0.873)	n.s. (*p* = 0.195)
OS, yes or no *~	n.s. (*p* = 1.0)	n.s. (*p* = 0193)	n.s. (*p* = 0.114)	n.s. (*p* = 0.195)
DSS, yes or no *~	n.s. (*p* = 1.0)	n.s. (*p* = 0.193)	n.s. (*p* = 0.339)	n.s. (*p* = 0.195)
NED/DFS, yes or no *~	n.s. (*p* = 0.114)	n.s. (*p* = 0.193)	***P* = 0.006**	***p* = 0.013**
Advanced tumors, yes vs. no *	***p* = 0.001**	***p* = 0.0001**	n.s. (*p* = 1.0)	***p* = 0.001**
Hyams grading III–IV, yes vs. no *	n.s. (*p* = 0.628)	n.s. (*p* = 0.255)	n.s. (*p* = 1.0)	n.s. (*p* = 0.756)
Negative margins rate, yes vs. no *	n.s. (*p* = 1.0)	***p* = 0.022**	n.s. (*p* = 0.532)	n.s. (*p* = 1.0)
Complete resection rate (NM or GTR) *	n.s. (*p* = 0.601)	n.s. (*p* = 0.10)	n.s. (*p* = 0.560)	n.s. (*p* = 0.562)
Surgical complications, yes vs. no *	***p* = 0.015**	n.s. (*p* = 0.126)	n.s. (*p* = 0.145)	***p* = 0.019**
RT, yes vs. no *	n.s. (*p* = 0.153)	n.s. (*p* = 0.302)	n.s. (*p* = 1.0)	n.s. (*p* = 0.184)
ChT, yes vs. no *	***p* = 0.015**	***P* = 0.013**	n.s. (*p* = 1.0)	***p* = 0.022**
Recurrence, yes vs. no *	n.s. (*p* = 0.106)	n.s. (*p*= 0.338)	***p* = 0.031**	***p* = 0.034**

^#^Mann–Whitney U-test/Fisher’s exact test; *Chi-square exact test; ^+^no statistics: too few cases per group. ~ Values were calculated from (raw) material in reports with variable follow-up times.

BCR, bicoronal transection; CFR, craniofacial resection; ChT, chemotherapy; DFS, disease-free survival; DSS, disease-specific survival; ES, endoscopic surgery; NED, no evidence of disease; OpS, open surgery; OS, overall survival; RT, radiation therapy; TCR, transcranial resection; TFR, transfacial resection. Bold letters/values should highlight significant results.

#### 3.6.1 Studies/case series

In comparison with OpS ± E-ass, the mean follow-up period was significantly shorter after ES (*p*=0.048), mainly due to the longer time after TFR ± E-ass (*p*=0.034). Crude OS, DSS, and DFS rates were significantly higher after ES in comparison with OpS ± E-ass (all *p*=0.0001), CFR ± E-ass (DSS and DFS, *p*=0.0001 each), and TFR ± E-ass (DFS, *p*=0.047). Actuarial survival rates relative to the surgical approach were not identified in STUD/CS on BCR/TCR ± E-ass and TFR ± E-ass but were available for CFR ± E-ass and ES. For CFR ± E-ass, the actuarial 5-year OS was 60%–95.2%; for 5-year DSS, 77–82.6%; for 5-year DFS, 28.6%–86.5%; and for 5-year RFS, 49%–64.2%. In comparison, the actuarial survival after ES was higher, at 84.6%–100% for 5-year OS, 100% in the average for 5-year DSS, 50%–100% for 5-year DFS, and 75%–92.9% for 5-year RFS. After CFR ± E-ass, the 10-year survival rates were 42%–93% for OS, 53% for DSS, and 57.1% for DFS. The 15-year DFS reported in one publication was 82.6% (58). In comparison, after ES, the actuarial 10-year OS was 87.5%–100% and the 10-year DFS was 75.6%–90%.

**Figure 2:**
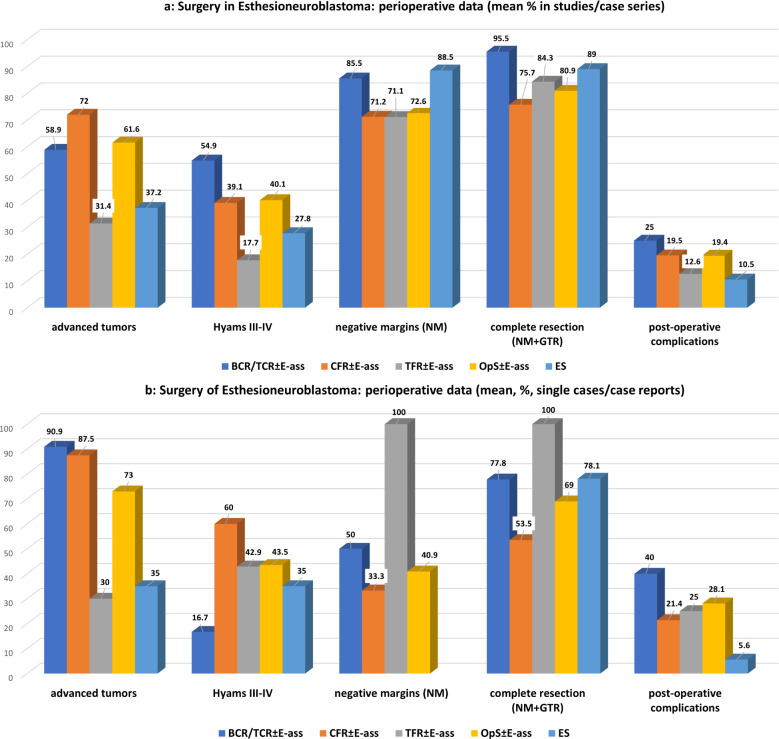
**(a)**Surgery for esthesioneuroblastoma: perioperative data of studies/case series for OpS±E-ass (BCR/TCR±E-ass, CFR±E-ass, TFR±E-ass) and endoscopic surgery (ES). Regarding significant differences, particularly, between BCR/TCR±E-ass, CFR±E-ass or OpS±E-ass and ES see **Tables 1a** and **2a**. **(b)** Surgery for esthesioneuroblastoma: perioperative data of single cases/case reports for OpS±E-ass (BCR/TCR±E-ass, CFR±E-ass, TFR±E-ass) and endoscopic surgery (ES). Regarding significant differences, particularly, between BCR/TCR±E-ass, CFR±E-ass or OpS±E-ass and ES see **Tables 1b** and **2b**.

**Figure 3:**
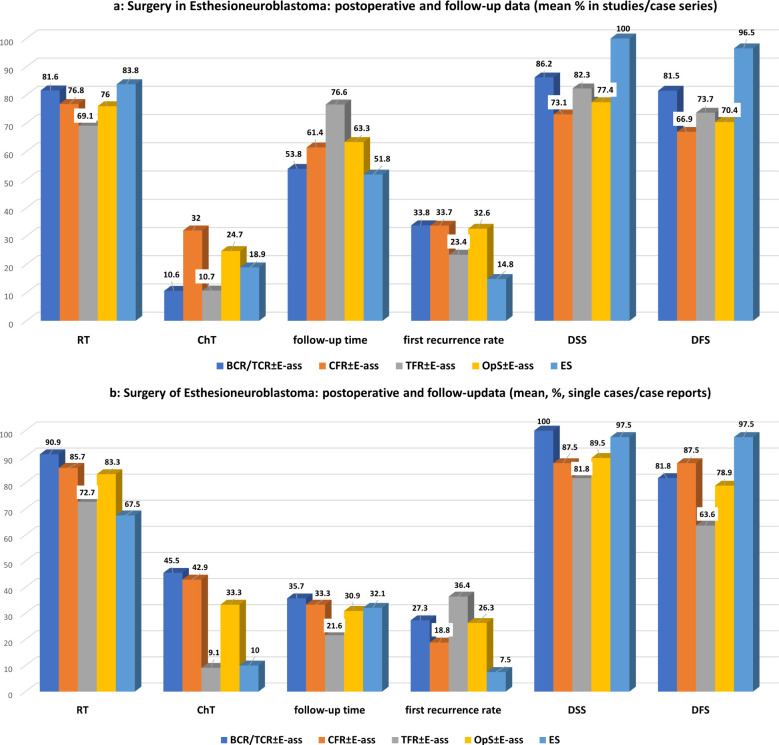
**(a)** Surgery for esthesioneuroblastoma: perioperative data of studies/case series for OpS ± E-ass (BCR/TCR ± E-ass, CFR ± E-ass, and TFR ± E-ass) and endoscopic surgery (ES). Regarding significant differences, particularly, between BCR/TCR ± E-ass, CFR ± E-ass, or OpS ± E-ass and ES, see **Tables 1a** and **2a**. **(b)** Surgery for esthesioneuroblastoma: perioperative data of single cases/case reports for OpS ± E-ass (BCR/TCR ± E-ass, CFR ± E-ass, and TFR ± E-ass) and endoscopic surgery (ES). Regarding significant differences, particularly, between BCR/TCR ± E-ass, CFR ± E-ass, or OpS ± E-ass and ES, see **Tables 1b**, **2b**.

Significantly more advance-stage tumors were treated with OpS ± E-ass, mainly due to the significantly larger number treated by CFR ± E-ass (*p*=0.0001 each). With regard to Hyams grade III–IV tumors, there was a tendency toward a higher frequency in ES in comparison with BCR/TCR ± E-ass and OpS ± E-ass cases, but no significant differences. After ES, higher rates of NMs were observed in comparison with OpS ± E-ass (*p*=0.009), and higher rates of NMs and CoRs were described compared to CFR ± E-ass (*p*=0.008 and *p*=0.036). Compared to BCR/TCR, CFR ± E-ass, and OpS ± E-ass, significantly lower rates of postoperative complications (*p*=0.022, *p*=0.046, and *p*=0.022) and fewer recurrences (*p*=0.036, *p*=0.0001, and *p*=0.0001) were described after ES. In addition, RT was administered after ES significantly less often compared to OpS ± E-ass (*p*=0.043) and ChT compared to CFR ± E-ass (*p*=0.041). In general, the differences were most significant when ES was compared to CFR ± E-ass (**Tables 1a**, **2a**; **Figures 2a**, **3a**).

#### 3.6.2 Single cases/CRs

In comparison with ES, DFS was significantly lower after TFR ± E-ass (*p*=0.006) and OpS ± E-ass (*p*=0.013), and significantly more advanced tumors were treated with BCR/TCR ± E-ass, CFR ± E-ass, or OpS ± E-ass (*p*=0.001, *p*=0.0001, and *p*=0.001). In addition to this, significantly more surgical complications were observed after BCR/TCR ± E-ass and OpS ± E-ass (*p*=0.015, *p*=0.019). Compared to ES, ChT was administered significantly more often after BCR/TCR ± E-ass, CFR ± E-ass, and OpS ± E-ass (*p*=0.015, p=0.013, *p*=0.022), and recurrence rates were significantly higher after OpS ± E-ass (*p*=0.034; **Tables 1b**, **2b**; **Figures 2b**, **3b**).

## 4 Discussion

This review compared the results after OpS ± E-ass, stratified relative to BCR/TCR ± E-ass, CFR ± E-ass, and TFR ± E-ass, and ES for esthesioneuroblastoma selecting 144 reports including 1,434 patients published from 1990 to 2023.

One early meta-analysis evaluated 26 STUD/CS published between 1990 and 2000 including 390 patients after unstratified OpS (12). Average rates of advance-stage tumors (Kadish stage C, 61%; T3–4, 50%) and Hyams grade III–IV tumors (38%), and recurrence rates (29% local, 16% regional, and 17% distant) were reported. Surgery and RT (dosages of 55–65 Gy) were performed in 44%. The average 5-year OS and 5-year DFS were 45% and 41%, respectively, and the 10-year OS was 52%. CFR was the most effective OpS, with a 5-year DFS of 65%. Compared to this, similar results were described in the STUD/CS reporting staged or combined/mixed OpS ± E-ass. Compared with results after CFR ± E-ass found in publications cited in this review, more cases were treated by surgery and RT (76.8%), and better 5-year OS (60%–95.2%) and DFS (28.6%–86.5%) rates and better and 10-year OS (42%–93%) survival rates were described (see *Results*; **Tables 1a**, **b**, **2a**, **b**; **Supplementary Tables S1**-**S4**).

ES for esthesioneuroblastoma was investigated in one recent review that included 44 STUD/CS and 399 patients. Reduced morbidity after ES ± RT was highlighted as most important advantages. Among the tumors, 48.3% had a modified Kadish stage C/D, and 34% were Hyams grade III–IV. NMs were achieved in 86.9%, and the mean recurrence rate was 10.3%. The reported mean 5-year survival rate was 91.1% (16). The results of the STUD/CS included in that review were comparable with those found in the present one. In the SC/CR, complete resection was achieved less frequently, presumably due to difficult locations. Nevertheless, low postoperative complication rates, low recurrence rates, and excellent survival rates were reported (**Tables 1a**, **b**; **Supplementary Tables 5A, B**).

Several meta-analyses and reviews comparing ES and OpS ± E-ass have been published. Devaiah et al. presented a meta-analysis including 361 patients treated from 1992 to 2008. Survival after ES was significantly better in comparison with OpS (100% vs. approximately 45%) or E-assisted OpS (100% vs. approximately 50%), also after the results had been stratified according to the publication year. OpS ± E-ass, 63%, was performed for Kadish stage C/D tumors, in comparison with 43.6% for ES. The median follow-up periods were similar for ES and OpS ± E-ass (54.5 vs. 51.0 months) (3).

Komotar et al. presented a review including 47 STUD/CS and 453 patients. Kadish stage A/B tumors were treated with ES significantly more often than with OpS ± E-ass. GTR was achieved in 98.1% of the patients in the ES group, in comparison with 81.3% after CFR ± E-ass and 100% after TCR; NMs were achieved in 93.8% after ES and 95.8% after TCR. The postoperative complication rates were lower after ES. The mean follow-up periods were 71 months after CFR ± E-ass, 52 months after ES, and 43.1 months after TCR ± E-ass. Local and regional recurrence rates were lower after ES in comparison with CFR ± E-ass or TCR ± E-ass (8.0% vs. 22.1% vs. 16.7%, and 6% vs. 17.3% vs. 8.3%). The 15-year OS and tumor progression-free survival according to Kaplan–Meier analysis were better after ES than after TCR ± E-ass and CFR ± E-ass (26).

Fu et al. evaluated 36 STUD/CS including 609 patients. The mean follow-up periods were 67.8 months for OpS ± E-ass and 52.4 months for ES. After ES, the postoperative complication rates (28.1% vs. 52.9%), frequency of locoregional recurrences (17.4% vs. 45%), distant metastases (1.1% vs. 7.5%), rates of cause-specific (0% vs. 15.2%), and overall mortality (0% vs. 19.9%) were all significantly lower in comparison with OpS ± E-ass. Although the Kadish stages were also significantly lower, more Hyams grade III–IV tumors were present in the ES group. After OpS ± E-ass, the median follow-up was 43 (1–312) months. The 5-year OS, DSS, locoregional recurrence-free survival (LRFS), and metastasis-free survival (MFS) rates were 71.2%, 77.5%, 78.8%, and 87.3%, and the 10-y OS, DSS, LRFS, and MFS rates were 57.0%, 72.7%, 61.7%, and 84%. The median follow-up period in the ES group was 32.5 (3–147) months. The 5-year OS, DSS, LRFS, and MFS rates were 100%, 100%, 79.5%, and 89.8%, respectively, and the 10-year OS, DSS, LRFS, and MFS rats were 100%, 100%, 69.6%, and 89.8%, respectively (25).

De Bonnecaze et al. evaluated 24 publications including 283 patients and 15 own patients. After surgery for advance-stage tumors, the highest survival rates were obtained after ES, including over the longer-term course. The 5-year OS rates were 95.8% after ES, 62.5% after OpS+E-ass, and 60.9% after OpS-E-ass (168).

Barinsky et al. reviewed 533 patients from the National Cancer Database; 51.8% underwent OpS ± E-ass and 48.2% ES. In the ES group, 53.2% of the tumors had Kadish stage C/D stages. After ES, the 5-year OS was 81.9% in comparison with 75.6% after OpS ± E-ass; a trend toward better survival after ES was observable after multivariate analysis (27).

The present systematic review is the first in which ES was compared to OpS ± E-ass consistently stratified into BCR/TCR ± E-ass, CFR ± E-ass, and TFR ± E-ass. It similarly showed significant differences between the results with ES and OpS ± E-ass for nearly all of the parameters tested—more for CFR ± E-ass than for BCR/TCR ± E-ass or TFR ± E-ass, and also more in STUD/CS than in SC/CR. The mean of the average follow-up times was significantly lower after ES in comparison with OpS ± E-ass (STUD/CS, *p*=0.048), mainly due to the differences compared to TFR ± E-ass (STUD/CS, *p*=0.034). This is not surprising, as ES was introduced more than 20 years later than all of the OpS ± E-ass with a measurable shift toward ES recognizable during the last years (**Tables 1a**, **b**, **2a**, **b**; **Supplementary Tables S1**-**S5B**). The frequency of advanced tumors treated was lower after ES, in particular if compared to OpS ± E-ass or CFR ± E-ass cases (STUD/CS, *p*=0.0001 and *p*=0.0001; SC/CR, *p*=0.0001, *p*=0.001) or BCR/TCR ± E-ass cases (SC/CR, *p*=0.001). Advance-stage tumors were operated on most often using CFR ± E-ass. Similarly, the proportion of STUD/CS in which >50% advance-stage tumors were present was highest for CFR ± E-ass but lowest and nearly equal for TFR ± E-ass and ES. These data reflect the fact that CFR ± E-ass, as the approach with the greatest invasiveness, is reserved for advance-stage ENBs. Interestingly, if the data were stratified according to the surgical approach, high-grade tumors were not significantly different distributed between ES and all OpS ± E-ass. Hyams grading, although recognized as an important prognostic factor (22, 23, 169–171), was not adequately addressed in many of the publications cited in this review. The available data support the view that its impact on the choice of surgical approach is limited. The appropriateness of the indication for the adequate surgical approach appears to be more dependent from tumor stage than Hyams grading. Of course, these interrelations should be investigated more intensively in the future (**Tables 1a**, **b**; **Supplementary Tables S1**-**S5B**). After ES, rates of NMs were significantly higher compared to CFR ± E-ass (STUD/CS, *p*=0.008) and OpS ± E-ass (STUD/CS, *p*=0.009), rates of total complete resection were significantly higher compared to CFR ± E-ass (STUD/CS, *p*=0.036)—results that also seem to point more toward the lower numbers of advanced tumors than high-grade tumors treated. Nevertheless, the literature also underscores the advantages of ES for advanced tumors reported in some STUD/CS (14, 26, 27, 65, 116, 118, 123, 129, 144, 160, 172–175). NMs were not achieved in single SC/CR, presumably due to very unusual or difficult locations. The importance of NMs was given greater importance in some reports than the surgical approach selected (14, 25, 27, 65, 174).

Surgical complication rates were significantly higher after BCR/TCR ± E-ass (STUD/CS, *p*=0.022; SC/CR, *p*=0.040), CFR ± E-ass (STUD/CS, *p*=0.046), and all OpS ± E-ass (STUD/CS, *p*=0.022) in comparison with ES, which may indicate that surgery in combination with craniotomy in particular carries a higher risk for postoperative complications. Compared to ES, RT was applicated with significantly lower frequencies compared to all OpS ± E-ass cases (STUD/CS, *p*=0.034), but not compared to the different OpS ± E-ass approaches. ChT was administered significantly more often after CFR ± E-ass (STUD/CS, *p*=0.029; SC/CR, *p*=0.013) and BCR/TCR ± E-ass (SC/CR, *p*=0.015). These data seem to reflect the higher rates of advance-stage tumors and a more complex surgical situation, particularly in cases treated with CFR ± E-ass. Nevertheless, although the rates of NMs were higher, the frequencies of RT/ChT in the ES patients were higher in comparison with OpS ± E-ass cases, possibly because ES was initially regarded as a new technique, and advance tumors were also resected with it as it emerged. The same causes may be involved in relation to recurrence rates. Rates of recurrences per study were significantly lower after ES in comparison with OpS ± E-ass (STUD/CS, *p*=0.0001; SC/CR, *p*=0.034), CFR ± E-ass (STUD/CS, *p*=0.0001), and BCR/TCR ± E-ass (STUD/CS, *p*=0.036), and in comparison with TFR ± E-ass (SC/CR, *p*=0.031). Notably, the time ranges after which the first recurrences developed were comparable in all groups. ES showed a favorable outcome in relation to survival rates. In comparison with OpS ± E-ass, crude OS and DSS (STUD/CS, *p*=0.0001 each), and DFS (STUD/CS, *p*=0.0001; SC/CR, *p*=0.013) were significantly better after ES. When ES was compared with the different OpS ± E-ass approaches, the most significant differences in DSS or DFS were observed after CFR ± E-ass (STUD/CS, *p*=0.0001 each) and BCR/TCR ± E-ass (STUD/CS, *p*=0.013, *p*=0.035). The 10-year actuarial survival reported, available only for ES and CFR ± E-ass, was higher after ES, at 87.5%–100% for OS and 75.6%–90% for DFS in comparison with 42–93% for OS, 53% for DSS, and 57.1% for DFS after CFR ± E-ass. In one publication, the 15-year DFS for CFR ± E-ass was 82.6% (58), with no comparable data for ES. In general, the results were somewhat more favorable in SC/CR, possible pointing to the fact that cases with specific characteristics and/or a favorable outcome were published. DFS after ES was significantly better compared to OpS ± E-ass and particularly to TFR ± E-ass (*p*=0.013, *p*=0.006; **Tables 1a**, **b**, **2a**, **b**; **Supplementary Tables S1**-**S5B**). The superior data published after ES may reflect the superior visualization provided by the magnification and ankle view of the endoscopes.

Overall, the data obtained in this review show that the results after ES are at least equivalent to OpS ± E-ass approaches in patients with ENB (**Tables 1a**, **b**, **2a**, **b**; **Figures 2a, b**, **3a, b**). ES was introduced 20 years ago, and advanced tumors were initially operated on less often using the technique. It was later reported that ES alone can achieve CoR even for more advanced tumor stages, provided that limitations are recognized and respected (14, 26, 27, 65, 116, 118, 123, 129, 144, 160, 172–175). In one report, the highest survival rates after surgery for advanced tumors were obtained after ES even over a longer-term course, with a 5-year OS of 95.8% (168). In another, it was found that NMs were achieved significantly more often after ES (84.2%) in comparison with OpS ± E-ass (52.1%) (14). The indication for ES is established mainly depending on the local extent of the tumor, and this is highlighted in most publications addressing ES and in those comparing ES with OpS ± E-ass (3, 25–27, 65, 168, 174). Growing experience with ES is reflected in the fact that conversion from ES to OpS ± E-ass was described in single cases up to the year 2010 (32, 111, 113, 123) but not after that (**Supplementary Tables 5A**, **5B**). Extended endoscopic endonasal transtuberculum/transplanum approach (EEA-TTP), as mentioned in the therapy of benign conditions (176), may represent the limit for ENB with cranial extension. As ENB is a malign tumor, it may be necessary, even after an extended ES has been performed, to supplement it by an open approach with or without endoscopic assistance (craniotomy ± E-ass) due to difficulties to achieve negative margins and the risk for massive complications caused by tumor infiltration of important/vital anatomical structures.

In this context, it has to be mentioned that new development in the radiation therapy, namely, by the introduction of radio-enhancers or peptide receptor radionuclide therapy, might influence the surgical decision making in these tumors, in particular in cases in which negative margins are expected to be difficult or not to achieve. Whether a major operative trauma could be avoided by applying a less-invasive surgical procedure, followed by radiation therapy with radio-enhancers or peptide receptor radionuclide therapy, which is sparing surrounding healthy cells, might be one of the most interesting topics for future research (177, 178).

Limitations of the review are the heterogeneity of the studies regarding patient number, design, follow-up time, and the report of prognostic histopathological factors (e.g., Hyams grading, Ki-67), resection state, surgical complications, details of the adjuvant therapy, and recurrence rates. Not all parameters of interest were included in every case series/study or report of single cases.

## 5 Conclusion

The data presented in this review support the conclusion that ES may be regarded as the surgical method of first choice for ENBs with Kadish stages A–B/T1–2. If limitations are respected, ES may be also a possible alternative in carefully selected advanced ENBs with Kadish stage C/T3 (14, 26, 27, 65, 116, 118, 123, 129, 144, 160, 172–175). BCR/TCR ± E-ass and CFR ± E-ass, in particular, are the surgical approaches of choice if the extent of an ENB exceeds the limits in terms of cranial extension (Kadish stage C/T4—e.g., brain, skull base, and optical nerve) and/or caudal extension (orbit and maxillary bone) (2, 31, 58, 179, 180). TFR ± E-ass is reserved for ENBs that mainly have an increased caudal extension (e.g., orbit, bone of nasal floor, or maxilla) (21, 88, 94, 95). In many cases, it is clear that an adequate surgical treatment, in particular (extended) ES or combined approaches, are associated with the best success rates if an adequate setting/skillset is available and an interdisciplinary team (ENT, neurosurgery, and maxillofacial) is involved.

The clinical implications of findings found in this review for practitioners are that these tumors can be treated successfully by (extended) ES in a substantial part of the cases. In extended tumor growth, open approaches with or without E-ass are indicated. Consequently, such cases should be managed by a multi-disciplinary team in high-volume units.

## Data Availability

The original contributions presented in the study are included in the article/**Supplementary Material**. Further inquiries can be directed to the corresponding author.
