# Supplementary material for: Endoscopic surgery versus various open approaches in esthesioneuroblastoma: a systematic review of the literature
Source: Front Oncol. 2025 May 28;15:1512771. doi: 10.3389/fonc.2025.1512771 (PMC12151833; doi:10.3389/fonc.2025.1512771)
Supplement: Supplementary file 1 [file Table1.docx]

**Supplemental Table 1.**Literature Data for Open Surgery: Bicoronal Resection/Transcranial Resection (BCR/TCR), Without or With Endoscopic Assistance.

| **Open surgery (all techniques)** | **Parameters** | | | | | | | | | | |
| --- | --- | --- | --- | --- | --- | --- | --- | --- | --- | --- | --- |
|  | Patients (n) with surgery (curative intent) | (Mean/median) follow-up (months) | Survival analysis | Advanced tumor stage; ectopic location/ unusual symptoms | Hyams grade III–IV | Negative margins/ GTR | Postoperative complication rate — only related to surgery (% of patients) | Pre/post-operative RT/SRT (%); (mean) dosage (range) or (mean) dosage ± SEM (Gy) | Pre/post-operative ChT (%) | (First) recurrence % (no. of patients; location); after (average/median) time and range or mean ± SEM (months) | Progression of primary tumor |
| **BCR/TCR** |  |  |  |  |  |  |  |  |  |  |  |
| Berman (1992) ^30^ | 1 | 24 | NED/DFS | Kadish C;  (sphenoid) | n.n. | n.n.;  Yes | No | Yes | Yes | No | No |
| Constantinidis (2004) ^2^ | 15 | 71.9 (4–194) | NED/DFS 73.3% DSS 73.3% | Mod. Kadish C/D 53.3% | 40% (6/13) | n.n. | 13.3% | 80%;  55 (50–65) | 6.7% | 26.7% (2 local + regional, 1 distant, 1 local + distant); 8, 10, 7, 21 | 6.7% |
| Wang (2005) ^31^ | 8 | 22 (1.3–100.8) | OS 87.5% DSS 100% DFS 75% | Kadish C 87.5% | n.n. | 100% | 25% | 100%; n.n. (55–65) | None | 12.5% (1 regional + distant); 1 + 3 | None |
| Poetker (2005) ^32^ | 1 | 102 | NED/DFS | Kadish C T4 | n.n. | No | No | Yes | Yes | Yes (local); 26 | No |
| Josephs (2008) ^33^ | 1 | 6 | NED | Kadish C; (Cushing Syndrome) | n.n. | n.n.;  Yes | No | Yes; 60  n.n. | No | No | No |
| Ward (2008) ^34^ | 15 | 75 (2-240) | OS 100%, NED/DFS 73.3%  5-y/15y DFS 49%/24%; 5y/15y RFS 49%/24% | Kadish C 13.3% | 86.7%; | n.n. | ≥30% | 40%; n.n. | 6.7% | 53.3% (5 local, 1 regional, 2 local + regional); 82.1 (n.n.) | None |
| Schmalisch (2009) ^35^ | 1 | 7 | NED/DFS | Kadish C; (sellar region) | n.n. | No | No | Yes; 53.2 | No | No | No |
| Mohindra (2014) ^36^ | 3 | 35  (47, 21, 37) | DSS 66.7% | Kadish C 66.7% | 66.7% | n.n. | 33.3% | 100%;  60 (60) | 33.3% | 66.7% (2 local) | None |
| **Endoscopy-assisted BCR/TCR** | | | | | | | | | | | |
| Devaiah (2003) ^37^ | 7 | 62.3  (24–96) | OS 100% DSS 100% DFS 85.7% | Kadish C 42.3% | 14.3% | n.n.; 100% | 42.3% | 71.4%;  n.n. | 16.7% | 28.6% (1 regional,1 local); 78, 59 | None |
| Zafereo (2008) ^38^ | 1 | 14 | NED/DFS | Kadish C T3 | No | Yes | Yes | Yes | Yes | No | No |
| Sham (2014) ^39^ | 1 | 48 | NED/DFS | Kadish C | n.n. | n.n. | No | Yes; 60 | No | No | No |
| Valdes (2014)  ^40^ | 1 | 14 | NED/DFS | Kadish C | No | n.n.;  Yes | Yes | Yes; 60 | No | No | No |
| Tomio (2014) ^41^ | 2 | 26.5 (8, 45) | NED/DFS 100% | Kadish C 100% | n.n. | n.n.; 100% | No | 100%;  n.n. | No | No | No |
| Buohliqa (2016) ^42^ | 1 | 98 | AWD | Kadish C | No | n.n. | n.n. | Yes; 60 | Yes | Yes (local); 29 | No |
| Chung (2020) ^43^ | 1 | 12 | NED/DFS | Kadish C | No | n.n.;  yes | No | Yes; 55 | No | No | No |
| Turri-Zadni (2021) ^28^ | 1 | 60 | NED/DFS | No; (anterior ethmoid) | No | Yes | Yes | Yes;  n.n. | No | No | No |
| McAvoy (2023) ^44^ | 1 | 8 | AWD | Kadish C | Yes | n.n.;  yes | Yes | No | Yes | Yes (distant); 2 ~ | Yes ~ |
| **BCR/TCR ± endoscopic assistance** | | | | | | | | | | | |
| Wertz (2018) ^45^ | 35 | 84 (6–252) | OS 90.9% DSS 90.9%; (5-y, 10-y, 15-y OS 97%, 92%, 83%^) *^ | Kadish C 49% | n.n. | 71%;  11% | 31% | 80%; n.n. | n.n. | 49% (n.n.); 29% > 60 months | none |

* Data refer to all patients in the publication (various surgical approaches included).

~ one possible case in this case report^44^, but not explicitly described as tumor progression

AWD, alive with disease; BCR, bicoronal resection; ChT, chemotherapy; DFS, disease-free survival; DSS, disease-specific survival; GTR, gross total resection; n.n.: no or no adequate data available; NED, no evidence of disease; OS, overall survival; RT, radiotherapy; SEM, standard error of the mean; SRT, stereotactic radiotherapy; TCR, transcranial resection.
