# Supplementary material for: Endoscopic surgery versus various open approaches in esthesioneuroblastoma: a systematic review of the literature
Source: Front Oncol. 2025 May 28;15:1512771. doi: 10.3389/fonc.2025.1512771 (PMC12151833; doi:10.3389/fonc.2025.1512771)
Supplement: Supplementary file 2 [file Table2.docx]

**Supplemental Table 2.** Literature Data for Open Surgery: Craniofacial Resection (CFR) Without or With Endoscopic Assistance.

| **Open surgery (all techniques)** | **Parameters** | | | | | | | | | | |
| --- | --- | --- | --- | --- | --- | --- | --- | --- | --- | --- | --- |
|  | Patients (n) with surgery (curative intent) | (Mean/median) follow-up (months) | Survival analysis | Advanced tumor stage; ectopic location/ unusual symptoms | Hyams grade III–IV | Negative margins/ GTR | Postoperative complication rate — only related to surgery (% of patients) | Pre/post-operative RT/SRT (%); (mean) dosage (range) or (mean) dosage ± SEM (Gy) | Pre/post-operative ChT (%) | (First) recurrence % (no. of patients; location); after (average/median) time and range or mean ± SEM (months) | Progression of primary tumor |
| **CFR** |  |  |  |  |  |  |  |  |  |  |  |
| Dulguerov (1992) ^21^ | 6 | 23.3 (7–15) | NED/DFS 100% | Kadish C 33.3% | n.n. | n.n. | n.n. | 83.3%;  n.n. (55–65) | None | 16.7% (1 local); 120 | None |
| Morris (1994) ^46^ | 2 | 30 (24, 36) | NED/DFS 100% | Kadish C 100% | n.n. | n.n.; 100% | n.n. | 50%; n.n. | None | None | None |
| Austin (1996) ^47^ | 6 | 65.1 (0.1–112) | NED/DFS 50% DSS 66.7% | T3/4 83.3% | n.n. | n.n. | 16.7% | 83.3%; n.n. | 16.7% | 66.7% (4 local); 3, 22, 39, 62 | None |
| Irish (1997) ^48^ | 9 | 43.4 (10–110) | NED/DFS 66.7% DSS 88.9% | Kadish C 66.7% | n.n. | n.n. | 11.1% | 88.9%; n.n. | 22.2% | 33.3% (1 local, 1 regional, 1 local + regional); n.n. | None |
| Sharma (1999) ^49^ | 6 | 38 (5-54) | NED/DFS 50%  OS 66.7%  DSS 66.7% | Kadish C 100% | n.n. | n.n. | n.n. | 100%; 54.3 (40-60) | 50% | 66.7% (1 local, 3 distant); 1, 3, 5, 24 | 16.7% |
| Miyamoto (2000) ^22^ | 8 | 42,3  (1–91) | NED/DFS 37.5% DSS 50% | Kadish C 75% | 37.5% | n.n. | n.n. | 75%; 67.25  (55.8–89.7) | 12.5% | 50% (2 local, 1 regional, 1 distant); 2, 23, 19, 46 | n.n. |
| Simon (2001) ^50^ | 7 | 66 (10–164) | NED/DFS 57.1%, OS 57.1%, DSS 71.4% | Kadish C 57.1% | n.n. | n.n. | n.n. | 71.4%; n.n. | 28.6% | 42.3% (2 local, 1 regional); 5, 51, 5 | None |
| Wei (2002) ^51^ | 1 | 24 | NED/DFS | No; (maxillary sinus) | No | No | No | No | No | No | No |
| Gondim (2002) ^52^ | 1 | 12 | NED/DFS | Kadish C | n.n. | n.n.; yes | No | Yes; 56 | Yes | No | No |
| Argiris (2003) ^53^ | 13 | n.n.;  [51.6 (7–218)^#^] | NED/DFS 46.1% DSS 61.5% | Kadish C 69.2% | 23.1% | n.n.; 84.6% | n.n. | 76.9%; 50–60 | 23.1% | 76.9% (4 local; 6 local + distant); 36.1 (2.5–216) | 15.4% |
| Lund (2003) ^54^ | 42 | 57 (2–206) | 5y- and 10y OS 61% and 42%; 5y- and 10y- DSS 77% and 53% | Kadish C 73% | n.n. | n.n. | 11.9% | 57%; 55–65 | 4.8% | 31% (7 local, 6 distant); local 37 (12–144); distant n.n. (3–144) | None |
| Dias (2003) ^55^ | 8 | 60 (18–124) | NED/DFS 75% DSS 87.5% | n.n. | n.n. | 87.5% | 37.5% | 78.5%; n.n. | None | 12.5% (distant); n.n. | 12.5% |
| Sampath (2006) ^56^ | 1 | 36 | NED/DFS | Kadish C | Yes | No; no | No | Yes;  60 | No | No | No |
| Bist (2006) ^57^ | 1 | 3 | NED/DFS | Kadish C | n.n. | n.n. | No | Yes;  60 | No | No | No |
| Loy & Levine (2006) ^58^ | 50 | 93 (1–330) | NED/DFS 66% 5- and 15-y DFS  86.5% and 82.6% | Kadish C 64% | n.n. | n.n. | 26% | 100% (36%/64%); n.n. | 64% | 34% (12 local + regional, 5 distant); 72 (n.n. - 120) | n.n. |
| Kim (2007) ^59^ | 8 | 45 (7–116) | NED/DFS 75% | Mod. Kadish C 87.5%; mod. Kadish D 12.5% | n.n. | n.n. | 25% | 62.5%; n.n. (40–54.3) | 12.5% | 50% (1 local, 1 regional, 1 distant, 1 loco-regional); n.n. | None |
| Nakao (2007) ^60^ | 6 | n.n.  [(6–325) ^#^] | NED/DFS 50% DSS 66.7% | Kadish C 66.7% | 33.3% | n.n. | n.n. | 66.7%;  n.n. | 50% | 83.3% (4 local, 1 regional); 3, 7, 51, 78, 70 | None |
| Kim (2008) ^61^ | 10 | 28.8 (3–116) | NED/DFS 30% DSS 50% | Kadish C 100% | n.n. | n.n. | n.n. | 100%; n.n. | 20% | 40%; n.n. | n.n. |
| Zafereo (2008) ^38^ | 12 | 84.7 (1–312) | NED/DFS 75% DSS 100% | Kadish C 50% | 20% | 83.3% | 25% | 83.3%; n.n. (45–70) | 25% | 25% (2 local, 1 regional); 88, 31, 45 | n.n. |
| Patel (2012) ^62^ * | 151 | 56 (1–123) | 5-y OS 77.7% 5-y DSS 82.6% 5-y RFS 64.2% | Kadish C 76.7% | n.n. | 67.6% | 32.5% | 81% (18-90);  (NA 34%; 50 (18–90);  Post-OP 47%; 58.7 (28–62)) | 6.0% | 29.2% (n.n.); n.n. | n.n. |
| Song (2012) ^18^ | 12 | 52 (n.n.) | 5-y DFS 41.7% NED 33.3% DSS 75% | Kadish C 100% | n.n. | n.n. | 33.3% | 75%; n.n. | 25% | 33.3% (n.n.); (3–143) | None |
| El Kababri (2014) ^63^ | 8 | 103.7  (47–197) | Alive/NED/DFS 100% | Kadish C 50% | n.n. | n.n. | n.n. | 87.5%; 56.9 (50–65) | 100% | None | None |
| Herr (2014) ^64^ | 22 | 73 (24–183) | 5- OS 95.2% 5-y DFS 86.4% | Kadish C 54.5% T3/4 63.6% | n.n. | 59.1% | 40.1% | 100%; 66.5 (54–70) | 36.4% | 27.3% (2 regional, 3 distant, 1 regional + distant); 44.5 (13–82) | None |
| Rimmer (2014) ^65^ | 65 | 90 (1–309) | 5-y/10-y OS 76.5%/67.8%; 5-y/10-y DFS 73.5%/57.1% | n.n. | n.n. | n.n. | n.n. | n.n. [(55–65)^#^] | Yes;  n.n. | n.n.; 32%/22 local; 51 (3–233) 21.5%/14 regional; n.n. 29.2%/19 distant/intracranial; 58 (1–139) | n.n. |
| Petruzzelli (2015) ^66^ | 20 | 107.3 (6.53–248.23) | NED/DFS 60% DSS 70% | Kadish C 65% | n.n. | 70% | 25% | 100%; NA 50,  postop. 60 (52–61.2) | 25% | 55% (3 regional, 4 distant, 1 local + distant, 3 regional + distant); 45.9 (3–168) | None |
| Niyaz (2015) ^67^ | 1 | 4 | DOD | Kadish C | n.n. | No | n.n. | Yes; n.n. | Yes | Yes (distant); 1 | Yes |
| Tajudeen (2015) ^68^ | 8 | 47.9 | n.n. | Kadish C 85.7% | n.n. | 75% | 62.5% | n.n.; 50–60 | None | n.n. | None |
| Kim (2016) ^69^ | 1 | 61 | NED/DFS | Kadish C | n.n. | No;  Yes | n.n. | Yes; 50 | No | Yes (distant); 60 | No |
| Meyer (2017) ^70^ | 1 | 12 | NED/DFS | Kadish C | Yes | Yes | No | Yes;  n.n. | Yes | No | No |
| Hwang (2017) ^71^ | 16 | 83.5 (11–360) | NED/DFS 31.3% 5-y DFS 28.6% | Mod. Kadish C 87.5%; mod. Kadish D 12.5% | n.n. | n.n. | n.n. | 81.3%;  n.n. [(45-68)^#^] | 43.8% | 68.8% (2 local, 3 regional, 1 local + regional, 3 local + distant, 2 regional + distant); 52.4 (9-312) | None |
| König (2018) ^72^ | 14 | 88.45 (23.2–215.56) | NED/DFS 64.3% DSS 71.4% 5-y and 10-y OS 70.7% each | Mod. Kadish C 57.1%; mod. Kadish D 7.1% | 35.7% | 64.3% | 14.3% | 85.7%;  n.n. (46–66) | 21.4% | 35.7% (2 local, 3 local + distant); local mean 10; distant mean 103 | None |
| Toader (2018) ^73^ | 1 | 20 | NED/DFS | Kadish C | Yes | n.n.;  Yes | No | Yes;  n.n. | Yes | No | No |
| Bartel (2018) ^74^ | 1 | 40 | DOD | Kadish C | Yes | No | No | Yes | Yes | Yes (local); n.n. | No |
| Dumont (2020) ^75^ | 7 | 84.7 (8–208) | OS 57.1% DFS 57.1% DSS 71.4% | Mod. Kadish C 57.1%; mod. Kadish D 14.3% | n.n. | 14.3% | n.n. | 85.7%;  56.1 (54–60) | 71.4% | n.n. | None |
| Meerwein (2021) ^76^ | 4 | 95.3 (10–222) | NED/DFS 100% | None | n.n. | 75%; 25% | None | None | None | None | None |
|  |  |  |  |  |  |  |  |  |  |  |  |
| **Endoscopy-assisted CFR** |  |  |  |  |  |  |  |  |  |  |  |
| Liu (2003) ^77^ | 2 | 12.5 (8, 17) | NED/DFS 100% | Kadish C 100% | n.n. | 100% | None | 50%; 60 | 100% | None | None |
| Kenmochi (2003) ^78^ | 1 | 132 | NED/DFS | Kadish C | n.n. | No | Yes | Yes; 65 | No | No | No |
| Yuen (2005) ^79^ | 6 | 32.8 (12–72) | DFS 67% 5-y LRC 100% 5-y OS 60% | Kadish C 16.7% | n.n. | 100% | None | 33.3%;  n.n. | None | 33.3% (1 regional, 1 distant); 2, 15 | None |
| Vergani (2007) ^87^ | 1 | 12 | NED/DFS | Kadish C | No | Yes | No | n.n. | n.n. | No | No |
| Gendeh (2007) ^80^ | 1 | 3 | NED/DFS | Kadish C | n.n. | n.n.; yes | No | n.n. | n.n. | No | No |
| Radotra (2010) ^81^ | 1 | 14 | NED/DFS | Kadish C; (sellar region) | Yes | No | Yes | Yes; 60 | No | No | No |
| Aljumaily (2011) ^82^ | 1 | 8 | NED/DFS | Kadish C | Yes | Yes | No | Yes; 54 | Yes | No | No |
| Song (2012) ^18^ | 11 | 72 (n.n.) | NED/DFS 82% 5-y DFS 80.8% DSS 100% | Kadish C 72.7% | n.n. | n.n. | 9.1% | 100% | 90.9% | 9.1% (n.n.); n.n. | None |
| Yamamuro (2014) ^83^ | 1 | 18 | NED/DFS | Kadish C; (sellar region) | No | No | Yes | Yes; 18 | No | No | No |
| Tsang (2015) ^84^ | 14 | n.n. (17–188) | 5- and 10-y OS each 93%; 2y- and 5y-RFS 71% and 49% | Kadish C 64.3% | n.n. | 78.6% | 13.3% | 50%; n.n. | None | 50% (4 local, 3 regional); n.n. (3–47) | None |
| Bartel (2018) ^74^ | 3 | 44.7 (10, 39, 85) | NED/DFS 100% | Kadish C 100% | 66.7% | n.n. | None | 100%;  n.n. (60–66) | 100% | None | None |
| Meerwein (2021) ^76^ | 1 | 134 | NED/DFS | No | No | Yes | No | No | No | No | No |
|  |  |  |  |  |  |  |  |  |  |  |  |
| **CFR and endoscopy-assisted CFR** |  |  |  |  |  |  |  |  |  |  |  |
| Harvey (2017) ^14^ * | 42 | n.n.; [42.1 (6–421)^#^] | n.n.; 5-y DSS 85%^#^ 10-y DSS 63%^#^ | Kadish C 78.6% | 54.5% | 51.2% | n.n.; [16./%^#^] | 95.2%; 57.6 | Yes; n.n. | n.n.; [(local 12.8%^#^ regional 9.2%^#^ distant 15.6%^#^)]; n.n. | None |
| Kim (2019) ^85^ | 14 | n.n.; [53.8 (10.4-195.3)^#^] | 5-y PFS 35.9%^#^; 10-y OS 50%^#^ | Kadish C 92.9% | 42.9% | n.n.;  64.3% | n.n. | 78.6%; 55 (40-60) | 42.9% | 50% (2 local, 2 regional, 3 local + distant); n.n. | None |

* Multicenter study;

^#^ data refer to all patients in the publication (various therapies and/or surgical approaches included).

CFR, craniofacial resection; ChT, chemotherapy; DFS, disease-free survival; DOD, died of disease; DSS, disease-specific survival; PFS, progression-free survival; GTR, gross total resection; n.n.: no or no adequate data available; LRC, locoregional control; NED, no evidence of disease; OS, overall survival; RT, radiotherapy; SEM, standard error of the mean; SRT, stereotactic radiotherapy.
