# Supplementary material for: Endoscopic surgery versus various open approaches in esthesioneuroblastoma: a systematic review of the literature
Source: Front Oncol. 2025 May 28;15:1512771. doi: 10.3389/fonc.2025.1512771 (PMC12151833; doi:10.3389/fonc.2025.1512771)
Supplement: Supplementary file 3 [file Table3.docx]

**Supplemental Table 3.** Literature Data for Open Surgery: Transfacial Resection (TFR) Without or With Endoscopic Assistance.

| **Open surgery (all techniques)** | **Parameters** | | | | | | | | | | |
| --- | --- | --- | --- | --- | --- | --- | --- | --- | --- | --- | --- |
|  | Patients (n) with surgery (curative intent) | (Mean/median) follow-up (months) | Survival analysis | Advanced tumor stage; ectopic location/ unusual symptoms | Hyams grade III–IV | Negative margins/ GTR | Postoperative complication rate — only related to surgery (% of patients) | Pre/post-operative RT/SRT (%); (mean) dosage (range) or (mean) dosage ± SEM (Gy) | Pre/post-operative ChT (%) | (First) recurrence % (no. of patients; location); after (average/median) time and range or mean ± SEM (months) | Progression of primary tumor |
| **TFR** |  |  |  |  |  |  |  |  |  |  |  |
| Dulguerov & Calcaterra (1992) ^21^ | 13 | 114.8 (2–192) | NED/DFS 53.4% DSS 76.9% | Kadish C 15.4% | n.n. | n.n. | n.n. | 53.8%; n.n. (55–65) | 7.7% | 61.5 % (5 local, 2 local + regional, 1 distant); 26.8 (2–84) | None ~ |
| Austin (1996) ^47^ | 5 | 68.8 (8–225) | NED/DFS 60% DSS 100% | T3/4 20% | n.n. | n.n. | 20% | 80%; n.n. | None | 40% (2 local); 5, 9 | None |
| Irish (1997) ^48^ | 3 | 86.7 (96, 150, 20) | NED/DFS 100% | Kadish C 66.7% | n.n. | n.n. | n.n. | 66.7%; n.n. | None | None | None |
| Iliades (2002) ^88^ | 3 | 46 (12-108) | NED/DFS 100% | No | n.n. | n.n.;  100% | n.n. | 100%; n.n. (60; n.n.; n.n.) | None | None | None |
| Ghaffar (2005) ^89^ | 1 | 15 | NED/DFS | No | n.n. | n.n. | No | Yes;  60 | No | No | No |
| Unal (2006) ^90^ | 1 | 24 | NED/DFS | No;  (maxillary sinus) | No | Yes | n.n. | No | Yes | No | No |
| Kim (2007) ^59^ | 1 | 34 | DOD | Kadish D | n.n. | n.n. | Yes | Yes; n.n. | No | Yes (local); n.n. | No |
| Nakao (2007) ^60^ | 5 | n.n. [(6–325)^#^] | NED/DFS 40% DSS 60% | Kadish C 20% | 20% | n.n. | n.n. | 80%; n.n. | 20% | 60% (2 local, 1 distant); 14, 40, 17 | No |
| Papadogeorgakis (2009) ^91^ | 1 | 18 | NED/DFS | No (nasal cavity) | Yes | n.n.; yes | No | No | No | No | No |
| König (2018) ^72^ | 3 | 75.8 (100, 34.1, 93.4) | NED/DFS 33.3% OS 33.3% | Kadish C 66.7% | 33.3% | 33.3% | None | 66.7%; n.n. | 33.3% | 33.3%; n.n. | None |
| Von Zeidler (2014) ^92^ | 1 | 22 | AWD | n.n. ;  (oral cavity + nasal floor) | Yes | n.n. | No | Yes; 60 | No | Yes (local); 12 | No |
| Tajudeen (2015) ^68^ | 20 | 39 | n.n. | Mod. Kadish C 38.5%; mod. Kadish D 15.4% | n.n. | 80% | 40% | n.n.; 50–60 | 15.4% | n.n. | None |
| **Endoscopic-assisted TFR** |  |  |  |  |  |  |  |  |  |  |  |
| Prasad (2007) ^93^ | 1 | 12 | DOD | Kadish C | n.n. | Yes | No | No | No | Yes (local); n.n. (> 12) | No |
| Nabili (2011) ^94^ | 13 | 33.5 (11–64) | OS 92.3% DSS 92.3% NED/DFS 76.9% | T3 15.4% T4 30.8% | n.n. | 92.3% | 15.4% | 100%; n.n. | 30.8% | 15.4% (1 local, 1 regional); n.n. | None |
| Wormald (2011) ^95^ | 4 | 118.5 (18, 120, 60, 276) | NED/DFS 100% | Kadish C 25%; (max.+ sinus+ ant. ethmoid; sphenoid sinus; NP+PPF+ITF; nasal floor) | None | 50%; 50% | None | 75%; 60, 54, 60 | None | None | None |
| Dupuy (2012) ^96^ | 1 | 36 | AWD | No;  (maxillary sinus, oral cavity) | Yes | n.n. | n.n. | Yes; 60 | No | Yes (local); 12 | No |
| Kumar (2013) ^97^ | 1 | 10 | NED/DFS | No; (middle meatus) | n.n. | n.n.; | Yes | Yes;  n.n. | No | No | No |
| Vucinic ^98^ | 1 | 24 | NED/DFS | No | No | n.n.;  Yes | No | Yes;  60 | No | No | No |
|  |  |  |  |  |  |  |  |  |  |  |  |
| Jiang (2015) ^99^ | 1 | n.n. | NED/DFS | No; (maxillary sinus) | No | Yes | No | Yes; n.n. | No | No | No |
| Petruzzelli (2015) ^66^ | 2 | 106 (140.63, 178.43) | NED/DFS 100% | None | n.n. | 100% | None | None | None | None | None |
| Bartel (2018) ^74^ | 1 | 21 | NED/DFS | Kadish C | No | n.n. | n.n. | Yes; n.n. | No | No | No |
|  |  |  |  |  |  |  |  |  |  |  |  |

^#^ Data refer to all patients in the publication (various surgical approaches included);

~ one possible case in this case series (7.7%), but not explicitly described.

AWD, alive with disease; ChT, chemotherapy; DFS, disease-free survival; DOD, died of disease; DSS, disease-specific survival; GTR, gross total resection; n.n.: no or no adequate data available; NED, no evidence of disease; OS, overall survival; RT, radiotherapy; SEM, standard error of the mean; SRT, stereotactic radiotherapy.
