# Supplementary material for: Endoscopic surgery versus various open approaches in esthesioneuroblastoma: a systematic review of the literature
Source: Front Oncol. 2025 May 28;15:1512771. doi: 10.3389/fonc.2025.1512771 (PMC12151833; doi:10.3389/fonc.2025.1512771)
Supplement: Supplementary file 4 [file Table4.docx]

**Supplemental Table 4.** Literature data of Open Surgery: Combined (same case), staged (same case) or mixed OpS±E-ass (different cases).

| **Open surgery (all techniques)** | **Parameters** | | | | | | | | | | |
| --- | --- | --- | --- | --- | --- | --- | --- | --- | --- | --- | --- |
|  | Patients (n) with surgery (curative intent) | (Mean/median) follow-up (months) | Survival analysis | Advanced tumor stage; ectopic location/ unusual symptoms | Hyams grade III–IV | Negative margins/ GTR | Postoperative complication rate — only related to surgery (% of patients) | Pre/post-operative RT/SRT (%); (mean) dosage (range) or (mean) dosage ± SEM (Gy) | Pre/post-operative ChT (%) | (First) recurrence % (no. of patients; location); after (average/median) time and range or mean ± SEM (months) | Progression of primary tumor |
| **Several types of open surgery** | | | | | | | | | | | |
| Kutluhan (2008) ^100^: TCR+MFD | 1 | 36 | NED/DFS | Kadish C | n.n. | n.n.;  Yes | No | No | No | No | No |
| De Gabory (2011) ^101^: CFR, TCR, TFR | 26 | n.n.; [99 (12–360)^#^] | n.n.;  5-y & 20-y DSS 88.6% and 66.4%^#^; 5-y & 20-y DFS 84% and 29.8%^#^ | T3 30.8% T4 15.4% | n.n. | n.n. | 30.7% | 88.5%; 53.3 (45–60) | n.n. | 23.1% (4 regional, 1 local + regional, 1 local); 18, 72, 96, 132, 12, 204 | 7.7% |
| Bäck (2012) ^102^: TPR, TFR, TBR±E-ass, CFR | 15 | 78.2 (4–158) | NED/DFS 53.3% | Kadish C 66.7% | 26.7% | n.n.; 100% | 46.7% | 66.7%; 60 (45–60) | 13.3% | 46.7% (1 local, 1 local + distant, 5 local + regional + distant);  57 (6–110) | None |
| Belliveau (2016) ^103^: ES+DCS (2-staged) | 1 | 17 | NED/DFS | No;  (lacrimal sac) | No | n.n.;  Yes | No | Yes;  59.4 | No | No | No |
| _Ogawa (2020)_^104^_: TC+ES-assTCR_ | _1_ | _7_ | _NED_ | _Kadish C_ | _No_ | _No_ | _No_ | _Yes;_  _60_ | _No_ | _No_ | _No_ |

^#^ Data refer to all patients in the publication (various therapies and/or surgical approaches included): De Gabory et al. ^101^: two patients after nonsurgical approaches included: 1 patient DOD 12 months after palliative therapy, 1 patient NED after 60 months after RT with 70 Gy.

CFR, craniofacial resection; ChT, chemotherapy; DCS, dacryocystostomy; DFS, disease-free survival; DSS, disease-specific survival; E-ass, endoscopy-assisted; GTR, gross total resection; MFD, midfacial degloving; NED, no evidence of disease; n.n.: no or no adequate data available; RT, radiotherapy; SEM, standard error of the mean; SRT, stereotactic radiotherapy; TBR, transbasal resection, TCR, transcranial resection; TFR, transfacial resection; TPR, transpalatinal resection
