# Supplementary material for: Endoscopic surgery versus various open approaches in esthesioneuroblastoma: a systematic review of the literature
Source: Front Oncol. 2025 May 28;15:1512771. doi: 10.3389/fonc.2025.1512771 (PMC12151833; doi:10.3389/fonc.2025.1512771)
Supplement: Supplementary file 5 [file Table5.docx]

**Supplemental Table 5a.** Literature Data for Endoscopic Surgery (ES): Case Series, Studies.

| **Endonasal endoscopic surgery** | **Parameters** | | | | | | | | | | | |
| --- | --- | --- | --- | --- | --- | --- | --- | --- | --- | --- | --- | --- |
|  | Patients (n) with surgery (curative intent) | Additional craniotomy | (Mean/median) follow-up (months) | Survival analysis | Advanced tumor stage; ectopic location/ unusual symptoms | Hyams grade III–IV | Negative margins/ GTR | Postoperative complication rate — only related to surgery (% of patients) | Pre/post-operative RT/SRT (%); (mean) dosage (range) or (mean) dosage ± SEM (Gy) | Pre/post-operative ChT (%) | (First) recurrence % (no. of patients; location); after (average/median) time and range or mean ± SEM (months) | Progression of primary tumor |
| Casiano (2001) ^106^ | 5 | None | 31 (6–36) | OS 100% DFS 80% | None | n.n. | 100% | 60% | 100%; n.n. | None | 40% (1 regional + distant, 1 distant); 8, 36 | None |
| Constantinidis (2004) ^2^ | 6 | None | 125.2 (20–242) | DSS 100% | Kadish C 16.7% | None (0/3) | n.n. | 16.7% | 50%; n.n. (24–65) | None | None | None |
| Unger (2005) ^111^ | 14 | 2 | 58 (13–128) | DFS 100% | Kadish C 62.3% | 92.6% | n.n.; 100% | 7.1% | 100%; 15–34 | None | 35.7% (3 local, 2 distant); 36.6 (79, 46, 18, 34, 6) | None |
| Poetker (2005) ^32^ | 5 | 1 | 68 (38, 61, 78, 95) | NED/DFS 100% | Kadish C 25% | n.n. | n.n. | n.n. | 75%; n.n. | 25% | 25% (1 local); 3 | None |
| Xie (2007) ^113^ | 6 | 1 | 44.5 (14–63) | NED/DFS 83.3% | Kadish C 66.7% | n.n. | n.n. | n.n. | 83.3%; n.n. | None | 16.7% (1 local + distant); 31 | None |
| Suriano (2007) ^115^ | 9 | None | 42.8 (26–60) | NED/DFS 100% | None | n.n. | n.n.; 100% | 11% | 100%; 56 (40–60) | None | None | None |
| Castelnuovo (2007) ^116^ | 10 | None | 37 (15–79) | OS 100% DSS 100% DFS 100% | Kadish C 30% | 20% | 100% | None | 90%; 56.1 | None | None | None |
| Dave (2007) ^118^ | 10 | None | 40.5 (3–105) | Alive 100% DSS 100% | Kadish C 20% | n.n. | 100% | 30% | 90%; n.n. | None | 10% (1 regional); n.n. | None |
| Kim (2008) ^61^ | 9 | None | 20.7 (4–41) | NED/DFS 88.9% DSS 100% | Kadish C 77.8% | n.n. | n.n. | n.n. | 100%; n.n. | 88.9% | None | None |
| Li (2008) ^119^ | 9 | None | n.n. | 5-y OS 88.9% | Kadish C 22.2% | n.n. | n.n. | n.n. | 100%; n.n. | None | 33.3% (3 local); 16 (n.n.) | None |
| Zafereo (2008) ^38^ | 3 | None | 67.3 (21, 34, 147) | NED/DFS 100% | None | None | 66.7% | None | 33.3%; n.n. | None | 33.3% (1 local); 38 | None |
| Folbe (2009) ^123^ | 19 (after primary therapy) | 1 | 45.2 (11–152) | NED/DFS 100% | Mod. Kadish C 26.3%; mod. Kadish D 5.3% | n.n. | 89.5% | 31.6% | 84.2%; n.n. | None | None | None |
| Zhang (2010) ^124^ | 3 | None | 18.7 (18, 18, 20) | NED/DFS 100% | None | n.n. | n.n. | n.n. | 100%; n.n. (55–65) | None | None | None |
| Monteiro (2011) ^127^ | 4 | None | 14 (7–17) | OS 100% DSS 100% | Mod. Kadish C 50%  mod. Kadish D 50% | n.n. | 100% | None | 100%; n.n. | 25% | None | None |
| Carta (2011) ^129^ | 5 | None | 21 (n.n.) | 5-y DSS 100% 5-y DFS 100% | Mod. Kadish C 20%; mod. Kadish D 20% | 20% | n.n. | None | 100%; n.n. [(54–72)^#^] | None | None | None |
| Song (2012) ^18^ | 5 | None | 88 (84–104) | NED/DFS 100% 5-yDFS 100% | Mod. Kadish C 20% ; mod. Kadish D 20% | n.n. | n.n. | None | 100%; n.n. | None | None | None |
| De Bonnecaze (2014) ^133^ | 8 | None | 95 (24–198) | 5-y OS 87.5% 5-y DFS 75% 5-y RFS 75% | Mod. Kadish C 50%  mod. Kadish D 50% | 62.5% | n.n.; 100% | 12.5% | 100%; n.n. | 50% | 25% (2 regional); 22, 50 | None |
| Rimmer (2014) ^65^ * | 30 | None | 73 (3–143) | 5-/10-y OS   each 100% 5-/10-y DFS 96%/75.6% | n.n. | n.n. | n.n. | n.n. | n.n. (55–65) | Yes; n.n. | 20% (2 local, 4 regional);  11, 39 (local) n.n. (regional) | n.n. |
| Mohindra (2014) ^36^ | 6 | None | 37.7 (26–57) | DSS 100% | Mod. Kadish D 16.7% | 16.7% | n.n. | None | 100%; 60 (60) | 16.7% | 16.7% (local);  n.n. | None |
| Chang (2015) ^135^ | 5 | None | n.n. (18–115) | NED/DFS 100% | n.n. | n.n. | n.n. | n.n. | 60%; n.n. | 20% | None | None |
| Petruzzelli (2015) ^66^ | 9 | None | 67 (43–88) | NED/DFS 100% | None | n.n. | 100% | None | 77.8%; n.n. | None | None | None |
| Feng (2015) ^136^ | 24 | None | 44 (8–130) | 3-y OS 82% 3-y DFS 70.8% | Kadish C 62.5% | n.n. | 79.2% | 4.2% | 83.3%; n.n. (50–66) | 29.2% | 29.1% (5 local, 3 regional, 2 distant); n.n. | None |
| Lund (2015) ^137^ | 36 | None | 77.6 (6–162) | 5- and 10-y OS 97% each 5- and 10-y DFS 90% each | n.n. | n.n. | n.n. | n.n. | 91.7%; n.n. | 69.4% | 13.9% (3 regional, 1 distant, 2 local + regional); 10, 14, 18, 26, 39 | None |
| Yokoi (2015) ^140^ | 2 | None | 37 (50, 24) | NED/DFS 100% | None | None | 100% | None | 50%; 18 (18 each) | None | None | None |
| Tajudeen (2015) ^68^ * | 8 | None | 12.5 (n.n.) | n.n. | Mod. Kadish C 37.5% mod. Kadish D 12.5% | n.n. | 100% | 12.5% | n.n.; n.n. (50–60) | None | n.n. | None |
| Tajudeen (2016) ^141^ ^+^ | 14 | None | 51.7 (14–145) | NED/DFS 100% | Mod. Kadish C 42.9%; mod. Kadish D 14.3% | n.n. | 100% | n.n. | 100%; n.n. | 28.6% | None | None |
| Zhang (2016) ^143^ | 10 | None | 79.3 (23–116) | Alive/OS 60% | Mod. Kadish C 50%; mod. Kadish D 10%; T3/4 50% | 40% | 80% | n.n. | 90%; n.n. (55–65) | None | n.n. | None |
| Manthuruthil (2016) ^144^ | 10 | None | 21.1 (n.n.) | OS 90% DSS 100% DFS 90% | Kadish C 50% | n.n. | 90% | 20% | 100%; n.n. (60–67) | 70% | None | None |
| Soldatova (2016) ^145^ | 13 | None | 31.8 (4.5–58) | NED/DFS 76.9% DSS 100% | Mod. Kadish C 38.5%; mod. Kadish D 15.4%; T3 46.2% | 38.5% | n.n. | n.n. | 76.9%; n.n. | 7.7% | 15.4% (1 regional, 1 regional + distant); 33, 19 | None |
| Hwang (2017) ^71^ | 10 | None | 63.9 (13–165) | 5-y DFS 90% | Kadish C 20% | n.n. | 60% | n.n. | 80%; n.n. [(45–68)^#^] | 30% | 10% (1 local), 7 | None |
| Harvey (2017) ^14^ * ^+^ | 67 | None | n.n. [42.1 (6–421)^#^] | n.n. [5-y DSS 85%^#^, 10-y DSS 63%]^#^ | Kadish C 56.7% | 35.8% | 88.1% | n.n. [16./%^#^] | 77.6%; 56.6±8.6 | Yes; n.n. | n.n. (local 12.8%^#^, regional 9.2%^#^, distant 15.6%^#^); n.n. | None |
| Bartel (2018) ^74^ | 4 | None | 66.5 (41–107) | NED/DFS 100% 5-y DFS 100% | Kadish C 25% | 25% | 75% | n.n. | 50%; n.n. (60–66) | None | None | None |
| Wertz (2018) ^45^ * | 6 | None | 21.6 (4–48) | OS 100% DSS 100% DFS 100%;  [5-y 10-y, 15-y-OS 97%, 92%, 83%]^#^ | Kadish C 33.3% | n.n. | 83.3% | 16.7% | 83.3%; n.n. | n.n. | None | None |
| Klironomos (2018) ^149^ | 10 | None | 74.8 (6–120) | OS 100% DSS 100% | Mod. Kadish C 40%; mod. Kadish D 20% | 50% | 90% | 10% | 90%; n.n. (45–60) | None | 20% (2 local); 58, 43 | None |
| Nakagawa (2018) ^152^ | 22 | None | 44 (11–104) | 5-y OS 100% 5-y DFS 95.5% | Kadish C 59.1% | 9.1% | 95.5% | None | 90.9%; n.n. (50–60) | None | 4.5% (local); 12 | None |
| De Gabory (2018) ^153^ ^+^ | 53 | None | 45.4 ± 26.5 (n.n.) | 5-y OS 87% 5-y DFS 71% | Mod. Kadish C 47.2%; mod. Kadish D 13.2%; T3/T4 54.7% | 30.2% | 73.9% | 18.9% | 90.6%; 64.6 ± 5.3 | 32.1% | 18.9% (1 local, 3 regional, 5 distant, 1 local + regional + distant); 30.8±20.4 | n.n. |
| Gallia (2018) ^155^ | 20 | None | 71.3 (16–141) | 5-y OS 92.9% 5-y DSS 100% 5-y RFS 92.9% | Mod. Kadish C 60% mod. Kadish D 10% | 30% | 75% | 35% | 95%; n.n. | 25% | 10% (1 local, 1 regional); 43, 61 | None |
| Kim (2019) ^85^ | 14 | None | n.n.; [53.8 (10.4-195.3)^#^] | 5-y PFS 38.5%; 10-y OS 87.5% | Kadish C 7.1% | 14.3% | n.n.; 64.3% | n.n. | 57.1%;  55 (40-60) | 7.1% | 35.7% (3 local, 1 regional, 1 local + regional); n.n. | None |
| Martinez-Perez (2020) ^157^ | 2 | None | 103 (86, 120) | NED/DFS 100% | None | n.n. | 100% | None | 100%; n.n. | None | 100% (1 distant, 1 regional); 84, 60 | None |
| Dumont (2020) ^75^ | 4 | None | 61.3 (41–86) | 5-y DFS 50% | Kadish C 50% | n.n. | 50% | n.n. | 75%; 56, 56, n.n. , n.n. | 75% | n.n. | None |
| Turri-Zadni (2021) ^28^ | 2 | None | 18 (12, 24) | NED/DFS 100% | None (lacrimal sac, maxillary sinus) | None | 100% | None | 100%; 10+53.7, 62 | 50% | None | None |
| Meerwein (2021) ^76^ | 5 | None | 69.2 (12-109) | NED/DFS 100% | None | 50% (2/4) | 100% | None | None | None | None | None |
| Sun (2021) ^160^ | 26 | None | 42.3 (8-124) | 5-y OS 84.6%  5-y DFS 76.9% | Kadish C 100% | 50% | 92.3%; 7.7% | 7.7% | 96.2%;  n.n. (55-65) | 61.5% | 26.9% (3 local, 4 distant); n.n. | None |
| Lui (2023) ^29^ | 2 | None | 42 (60, 24) | NED 100% | None  (both maxillary sinus) | None | 100% | None | 100%;  (n.n., 52.2) | 50% | 50% (1local); 168 | None |

+ Multicenter study;

* publications with comparisons of endoscopic and open surgery;

[#] data refer to all patients in the publications (various surgical approaches included).

ChT, chemotherapy; DFS, disease-free survival; DSS, disease-specific survival; GTR, gross total resection; NED, no evidence of disease; n.n., no or no adequate data available; OS, overall survival; RFS, recurrence-free survival; RT, radiotherapy; SEM, standard error of the mean; SRT, stereotactic radiotherapy.
