# Supplementary material for: Endoscopic surgery versus various open approaches in esthesioneuroblastoma: a systematic review of the literature
Source: Front Oncol. 2025 May 28;15:1512771. doi: 10.3389/fonc.2025.1512771 (PMC12151833; doi:10.3389/fonc.2025.1512771)
Supplement: Supplementary file 6 [file Table6.docx]

**Supplemental Table 5b.** Literature Data for Endoscopic Surgery (ES): Case Reports.

| **Endonasal endoscopic surgery** | **Parameters** | | | | | | | | | | | |
| --- | --- | --- | --- | --- | --- | --- | --- | --- | --- | --- | --- | --- |
|  | Patients (n) with surgery (curative intent) | Additional craniotomy | (Mean/median) follow-up (months) | Survival analysis | Advanced tumor stage; ectopic location/ unusual symptoms | Hyams grade III–IV | Negative margins/ GTR | Postoperative complication rate — only related to surgery (% of patients) | Pre/post-operative RT/SRT (%); (mean) dosage (range) or (mean) dosage ± SEM (Gy) | Pre/post-operative ChT (%) | (First) recurrence % (No. of patients; location); after (average/median) time and range or mean ± SEM (months) | Progression of primary tumor |
| Roy (2000) ^105^ | 1 | No | 24 | NED/DFS | Kadish C; (sellar region) | n.n. | No | Yes | Yes; n.n. | No | No | No |
| Cakmak (2002) ^107^ | 1 | No | 24 | NED/DFS | No | n.n. | No; yes | No | Yes; 50 | No | No | No |
| Liu (2003) ^77^ | 1 | No | 30 | NED/DFS | No | n.n. | n.n.; yes | No | No | No | No | No |
| Pasquini (2003) ^108^ | 1 | No | 53 | NED/DFS | No | n.n. | n.n.; yes | No | No | No | No | No |
| Morris (2004) ^109^ | 1 | No | 15 | NED/DFS | Kadish C; (sphenoid + clivus) | n.n. | n.n.; yes | No | No | No | No | No |
| Mariani (2004) ^110^ | 1 | No | 25 | NED/DFS | Kadish C (sellar region) | Yes | n.n.; yes | No | No | No | No | No |
| Strek (2006) ^112^ | 1 | No | 12 | NED/DFS | No | n.n. | Yes | No | No | No | No | No |
| Lee (2007) ^114^ | 1 | No | 6 | NED/DFS | No; (inferior meatus) | Yes | Yes | No | Yes; n.n. | No | No | No |
| Kim (2007) ^59^ | 1 | No | 24 | NED/DFS | Mod. Kadish D | n.n. | n.n. | No | Yes; n.n. | No | No | No |
| Podboj (2007) ^117^ | 1 | No | 88 | NED/DFS | No | n.n. | Yes | No | No | No | No | No |
| Kodama (2009) ^120^ | 1 | No | 12 | NED/DFS | No; (pterygoid fossa) | No | Yes | No | Yes; 40 | No | No | No |
| Chan 2008) ^121^ | 1 | No | 16 | NED/DFS | Kadish C; (sphenoid+ clivus) | n.n. | n.n.; yes | No | Yes; 56 | No | No | No |
| Lin (2009) ^122^ | 1 | No | 12 | NED/DFS | Kadish C; (sellar region) | n.n. | n.n.; yes | No | Yes; 30 | No | No | No |
| Cho (2010) ^125^ | 1 | No | 25 | NED/DFS | No; (nasal septum) | No | Yes | No | No | No | No | No |
| Seccia (2010) ^126^ | 1 | No | 36 | NED/DFS | No; (PPF) | n.n. | n.n. | No | Yes; 65 | No | No | No |
| Jiang (2011) ^128^ | 1 | No | 21 | NED/DFS | Kadish C | n.n. | No | No | Yes; 66 | No | No | No |
| Akinfolarin (2012) ^130^ | 1 | No | < 12 | DOD | Kadish C; (sphenoid sinus) | Yes | No | n.n. | Yes; n.n. | Yes | Yes (distant); 5 | No |
| Simal (2012) ^131^ | 1 | No | 12 | NED/DFS | Kadish C | n.n. | Yes | Yes | Yes; n.n. | No | No | No |
| Senchak (2012) ^132^ | 1 | No | 24 | NED/DFS | No;  (SIADH) | No | Yes | No | No | No | No | No |
| Wessell (2014) ^134^ | 1 | No | 18 | NED/DFS | Kadish C; (smell preservation) | n.n. | Yes | No | No | No | No | No |
| El Kababri (2014) ^63^ | 1 | No | 120 | NED/DFS | No | n.n. | n.n. | No | Yes; 55 | No | No | No |
| Matsunaga (2015) ^138^ | 1 | No | 16 | NED/DFS | No | n.n. | Yes | n.n. | Yes; 60 | No | No | No |
| Uslu (2015) ^139^ | 1 | No | 36 | NED/DFS | Kadish C | No | No | No | Yes; 66 | No | Yes (regional); 24 | No |
| Leon-Soriano (2016) ^142^ | 1 | No | 48 | NED/DFS | No; (bilateral ethmoid) | No | n.n.; yes | No | Yes; 60 | No | No | No |
| Nakano (2017) ^146^ | 1 | No | 14 | NED/DFS | Kadish C; (SIADH) | n.n. | n.n. | No | Yes; 50 | Yes | No | No |
| Parilla (2017) ^147^ | 1 | No | 60 | NED/DFS | No;  (SIADH) | n.n. | n.n. | No | Yes; n.n. | No | No | No |
| Cante (2018) ^148^ | 1 | No | 24 | NED/DFS | No | No | No | No | Yes; 60 | No | No | No |
| Fosbol (2018) ^150^ | 1 | No | 3 | NED/DFS | No; (maxillary sinus) | No | No | No | Yes; n.n. | No | No | No |
| Maggiore (2018) ^151^ | 1 | No | 24 | NED/DFS | No (smell preservation) | No | Yes | No | No | No | No | No |
| Rasool (2018) ^154^ | 1 | No | n.n. | NED/DFS | No; (maxillary sinus) | No | n.n.; Yes | No | Yes; 60 | No | No | No |
| Wong (2019) ^156^ | 1 | No | 84 | NED/DFS | No; (maxillary sinus) | No | No | No | Yes; 50 | No | No | No |
| Al Osaimi (2021) ^158^ | 1 | No | 38 | NED/DFS | No | Yes | Yes | No | Yes; 60 | No | No | No |
| Zhong (2021) ^159^ | 1 | No | 96 | NED/DFS | Kadish C; (nasopharynx) | Yes | n.n. | No | No | No | Yes (local); 14 | No |
| Mims 2021 ^161^ | 1 | No | 6 | NED/DFS | No;  (maxillary sinus) | No | n.n. | No | Yes; n.n. | No | No | No |
| Heiland (2021)  ^162^ | 1 | No | 61 | NED/DFS | No;  (SIADH) | n.n. | Yes | n.n. | No | No | No | No |
| Pradana (2022) ^163^ | 1 | No | 15 | NED/DFS | No | n.n. | n.n. | No | Yes; n.n. | Yes | No | No |
| Kumaria (2022) ^164^ | 1 | No | 26 | NED/DFS | No;  (sphenoid sinus) | Yes | Yes | No | Yes;  60 | Yes; | No | No |
| Karp (2022) ^165^ | 1 | No | 36 | NED/DFS | Kadish C | Yes | Yes | No | Yes | No | No | No |
| Saad (2023) ^166^ | 1 | No | 24 | NED/DFS | No;  (SIADH) | No | Yes | n.n. | No | No | No | No |
| Saffaradeh (2023)  ^167^ | 1 | No | n.n. | NED/DFS | No;  (SIADH) | No | Yes | No | Yes | No | No | No |

ChT, chemotherapy; DFS, disease-free survival; DOD, died of disease; DSS, disease-specific survival; GTR, gross total resection; NED, no evidence of disease; n.n., no or no adequate data available; PPF, pterygopalatine fossa; SIADH, syndrome of inappropriate secretion of antidiuretic hormone; RT, radiotherapy; SRT, stereotactic radiotherapy.
